# Acyl chains stabilize the acylated domain and determine the receptor-mediated interaction of the Bordetella adenylate cyclase toxin with cell membrane

**DOI:** 10.1016/j.jbc.2025.110392

**Published:** 2025-06-19

**Authors:** Carlos Espinosa-Vinals, Jan Stransky, Radim Osicka, Adriana Osickova, David Jurnecka, Peter Sebo, Ladislav Bumba

**Affiliations:** 1 Institute of Microbiology of the Czech Academy of Sciences, Prague, Czech Republic; 2 Institute of Biotechnology of the Czech Academy of Sciences, Vestec, Czech Republic

**Keywords:** adenylate cyclase toxin, RTX toxin, protein folding, Bordetella pertussis, acylation

## Abstract

Acylated domains (ADs), like that of the *Bordetella pertussis* adenylate cyclase toxin (CyaA), are structures found in all pore-forming toxins from the family of Repeat-in-ToXin (RTX) proteins. These AD segments are fatty-acylated on ε-amino groups of conserved lysine residues, such as the K860 and K983 residues of CyaA. The ε-amide–linked acyl chains are essential for toxin activity and promote irreversible membrane insertion of the CyaA molecule, thus enabling the toxin to translocate its N-terminal adenyl cyclase enzyme domain into the host cell cytoplasm. In parallel, the membrane-inserted CyaA molecules can oligomerize into cation-selective pores in the plasma membrane. Here, we show that the attached acyl chains are not only crucial for membrane insertion of the toxin but also play an important role in CyaA folding. We demonstrate that assembly of the noncanonical **β**-roll structure in the C-terminal segment of the AD of CyaA is cooperatively directed by the Ca^2+^-driven folding of the adjacent RTX domain. In contrast, the N-terminal AD segment consists of an **α**-helical structure that folds independently of Ca^2+^ ion binding and may form one or two acyl binding site(s) accommodating the acyl chains protruding from the C-terminal AD segment. This acyl-mediated interaction between the N- and C-terminal segments promotes local structural rearrangements within the AD that significantly enhances the stability of the toxin molecule. These findings highlight the critical role of the acyl modification in membrane interaction capacity and structural stability of the CyaA toxin.

The adenylate cyclase toxin-hemolysin (CyaA, ACT, or AC-Hly) of pathogenic *Bordetella* species is a prototypic member of the Repeat-in-ToXins (RTX) family of leukotoxins (1, 2). The toxin plays a central role in virulence of the classical *Bordetella* species by targeting the complement receptor 3 (CR3), a CD11b/CD18 (α_M_β_2_) integrin heterodimer expressed on the surface of myeloid cells, such as macrophages, neutrophils, and dendritic cells (3, 4, 5, 6). With a low but well detectable activity, CyaA can also promiscuously bind to and penetrate a variety of host cells lacking CR3 (7). Upon binding, CyaA penetrates the plasma membrane of host cells and translocates its N-terminal adenylyl cyclase (AC) enzyme domain into their cytosol (8). Once inside the cell, the AC domain is activated by calmodulin and catalyzes uncontrolled conversion of cytosolic ATP into cAMP, thereby disrupting cellular signaling and quickly ablating the bactericidal functions of phagocytes (9, 10). Translocation of the AC domain occurs from cholesterol-rich lipid microdomains (lipid rafts) of cell membrane and is accompanied by an influx of extracellular calcium ions (11, 12). The influx of calcium further exacerbates cell membrane permeabilization by preventing the removal of toxin pores from cell membrane through the membrane repair/recycling machinery (13). As a result, the membrane-inserted CyaA molecules can permeabilize the plasma membrane of cells by forming small cation-selective (hemolytic) pores that enable potassium efflux from cells and can provoke colloid-osmotic (oncotic) cell lysis at higher toxin concentrations (14, 15).

The N-terminal AC domain (residues 1–400) of the 1706-residue long CyaA protein is linked to a C-terminal RTX hemolysin (Hly) moiety consisting of: (i) an “AC-to-Hly-linking segment” (residues 400–500) that facilitates the translocation of the AC domain, (ii) a hydrophobic domain (residues 500–700) responsible for pore formation, (iii) an acylated domain (AD, residues 700–1000) bearing amide-linked palmitoyl chains on lysine residues K860 and K983, (iv) an RTX domain (residues 1000–1638) involved in CR3 binding, and (v) a noncleavable C-terminal secretion signal (Fig. 1). The secretion signal is recognized by a type I secretion system (T1SS), which mediates the translocation of the CyaA polypeptide directly from the bacterial cytoplasm into the external environment in a single step across the bacterial cell wall (16, 17, 18). Low concentrations of calcium ions in the bacterial cytoplasm (<100 nM) maintain the negatively charged RTX moiety in an intrinsically disordered conformation (19, 20, 21), which is essential for engagement with and secretion of RTX substrates *via* the T1SS (22, 23). The movement of the transported polypeptide within the T1SS conduit is energized by ATP hydrolysis and proceeds through a “push-pull” mechanism comprising a Ca^2+^-driven cosecretional assembly of the RTX repeats into a β-roll structure that successively ratchets the extruding polypeptide (24).

**Figure 1:**
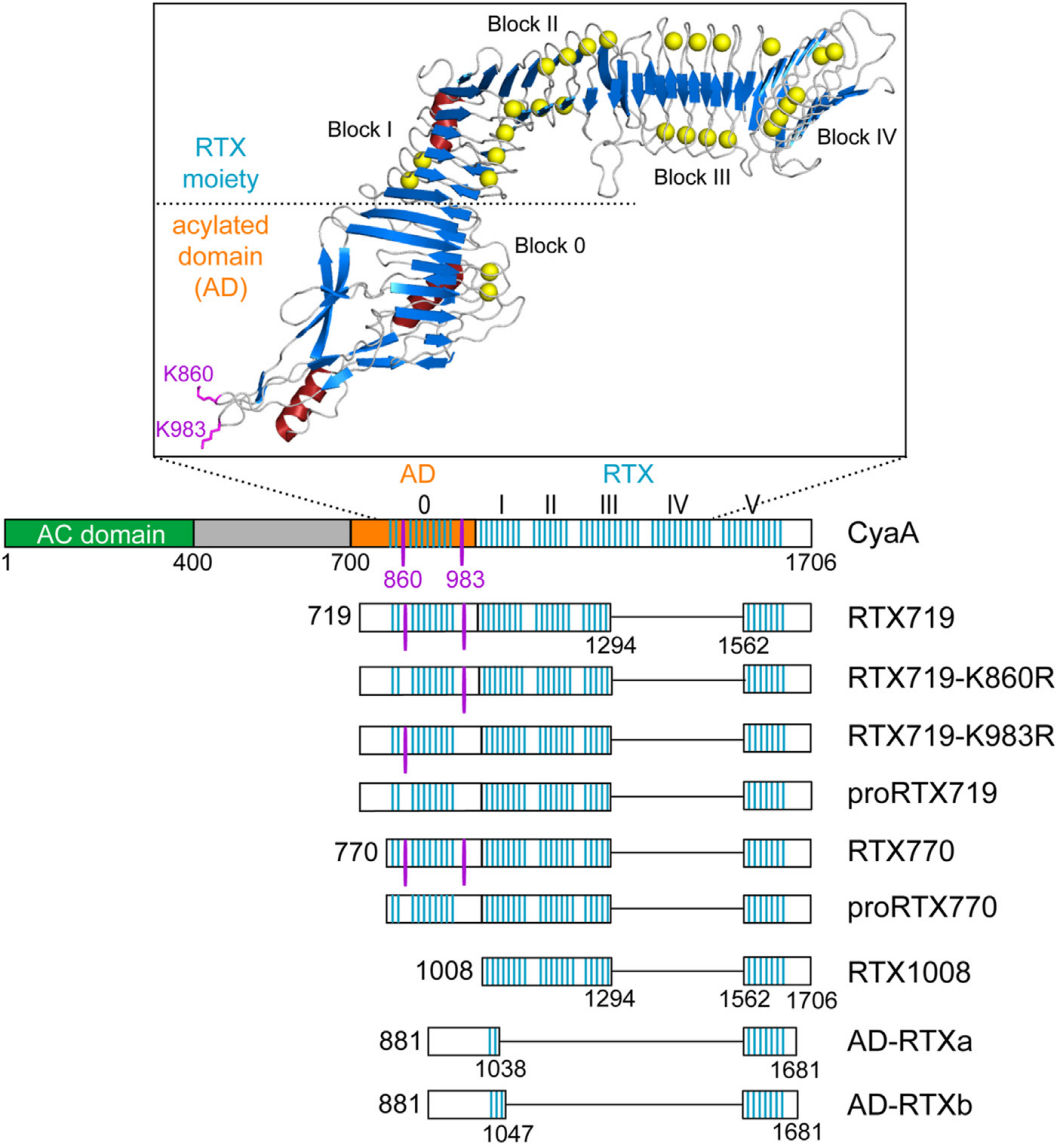
**Schematic representation of the adenylate cyclase toxin and CyaA-derived constructs used in this study**. CyaA consists of the N-terminal enzymatic adenylyl cyclase (AC) domain (*green*) linked to the C-terminal hemolysin moiety harboring the hydrophobic pore-forming domain (*gray*), the acylated domain (AD, in *orange*) with two acylation sites (K860 and K983, *magenta*), and the RTX domain constituted by five blocks of nonapeptide RTX repeats (I–V). The *blue bars* represent β-strands, which, within the RTX domain, are organized into a calcium-loaded parallel β-roll structure. The inset represents the high-resolution density map of the C-terminal RTX751 fragment of CyaA (residues 754–1488) obtained by cryo-EM (6). *Yellow balls* represent calcium ions. RTX, Repeat-in-ToXins.

CyaA is synthetized as an inactive protoxin (proCyaA) that is activated by covalent fatty acylation of the ε-amino groups of the internal lysine residues K860 and K983 by the coexpressed toxin-activating acyltransferase CyaC (25, 26). The CyaC acyltransferase selectively uses the 16-carbon acyl chain-loaded acyl–acyl carrier protein (ACP) as acyl chain donor for modification of the proCyaA substrate (27). While both lysine residues can be acylated, palmitoylation of the K983 residue alone is necessary and sufficient for CyaA binding to and penetration of the plasma membrane of CR3^+^ cells (28).

In recent years, low-resolution structural models of the RTX domain (21, 24) and the entire CyaA toxin molecule (29) have been determined using small angle X-ray scattering (SAXS). High-resolution structural data have also been obtained, including X-ray structures of the AC domain (30) and the RTX domain (31, 32), and an atomic model of the AC-to-Hly linking segment solved by nuclear magnetic resonance spectroscopy (33). Furthermore, a high-resolution density map of the nonacylated C-terminal CyaA fragment (residues 754–1488) in complex with the CR3 ectodomain was recently resolved using cryo-EM (6). This structure revealed that the AD of CyaA comprises a noncanonical β-roll (block 0), from which two long β-hairpins extend, bearing the K860 and K983 residues at their tips (Fig. 1). The AD is linked to the β-roll of the first RTX block I (RTX block I) by a linker segment, which is composed of an antiparallel β-strand helical assembly. Together, the AD and the RTX domain form a continuous β roll structure that assembles in a highly cooperative and calcium-driven folding process vectorially proceeding from the C terminus toward the N terminus of the polypeptide (24, 34).

In this study, we used truncated CyaA-derived constructs to explore the role of RTX repeats and acyl chains in the structural integrity of the AD. Our findings reveal that the Ca^2+^-driven folding of the RTX domain drives the folding of the AD and that acylation of the K860 and K983 residues significantly stabilizes its structure. Importantly, the acyl chains appear to be directly involved in promoting CR3-mediated insertion of CyaA into the membrane, playing a crucial role in folding and membrane insertion of the toxin.

## Results

### Calcium-driven folding of the RTX domain directs the folding of the AD of CyaA

**Figure 2:**
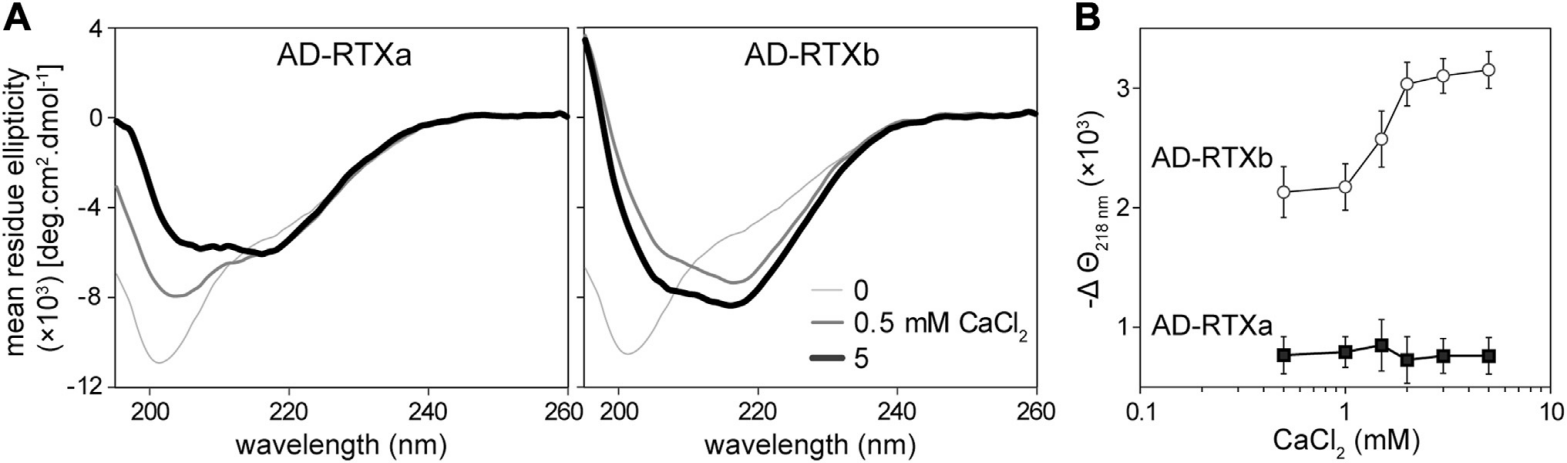
**Structural consecutiveness of the RTX repeats affects folding of the AD**. *A*, far-UV CD spectra of the AD-RTXa (*left panel*) and AD-RTXb (*right panel*) constructs in the absence (*thin line*), and the presence of 0.5 mM CaCl_2_ (*intermediate line*) and 5 mM CaCl_2_ (*thick line*). *B*, Ca^2+^-induced folding of the AD-RTXa (*black squares*) and AD-RTXb (*open circles*) constructs. The proteins (100 μg/ml) were titrated with increasing concentrations of CaCl_2_, and changes in the mean residue ellipticity were followed at 218 nm (Θ_218 nm_) as a function of Ca^2+^ concentration. The data represent mean values ± SD from three independent experiments. AD, acylated domain; RTX, Repeat-in-ToXins.

To investigate whether the folding of the AD is directed by the calcium-driven assembly of the RTX domain, we first examined the capacity of the adjacent β-roll in RTX block I to promote folding of the β-rich structure in the AD (Fig. 1). For this purpose, we designed two small AD-RTX constructs, AD-RTXa and AD-RTXb, with most of the RTX domain removed. The C-terminal folding scaffold of RTX block V (residues 1562–1681 of CyaA) was fused to one of the two CyaA segments comprising residues 881 to 1038 (AD-RTXa) or 881 to 1047 (AD-RTXb), respectively (Fig. 1). These constructs differ by a single nonapeptide stretch, as the presence or absence of one RTX nonapeptide was previously shown to influence the phase of cooperative folding within the β-roll structure (35). Both constructs were first expressed in the absence of the CyaC acyltransferase and purified as nonacylated proteins from a 8 M urea extracts of inclusion bodies under denaturing conditions. Upon dilution from the 8 M urea solution into nondenaturing Ca^2+^-free buffer, both AD-RTXa and AD-RTXb adopted an intrinsically disordered conformation, as evidenced by a strong negative band at 200 nm in their far-UV CD spectra (Fig. 2*A*). Calcium titration experiments revealed that the binding of Ca^2+^ ions induced structural transitions, leading to the formation of secondary structures at the expense of unordered conformations. However, striking differences in the secondary structure content between AD-RTXa and AD-RTXb emerged upon folding at a saturating calcium concentration of 5 mM. The AD-RTXa displayed high α-helix content with a limited amount of β-sheets, as indicated by two negative bands at 205 and 220 nm. In contrast, the far-UV CD spectrum of AD-RTXb exhibited a prominent negative band at 218 nm, typical for β-sheets. The Ca^2+^-induced formation of β-sheet structures in the AD-RTXb was clearly apparent from the calcium titration curve reflecting changes in the ellipticity at 218 nm as a function of calcium concentration (Fig. 2*B*). These findings suggested that the RTX linkage in the AD-RTXa construct was out of the β-roll folding phase, while the artificial RTXI/V block in the AD-RTXb construct enabled proper folding into a β-roll and the formation of a β-rich AD structure. Furthermore, the perturbed structural consecutiveness of the RTX repeats in the AD-RTXa construct resulted in protein misfolding, indicating that the folding of the AD proceeds cooperatively from the C terminus toward the N terminus in a process templated by the adjacent C-terminal RTX β-roll block.

### N-terminal segment of the AD forms a distinct subdomain structurally independent of the C-terminal segment

**Figure 3:**
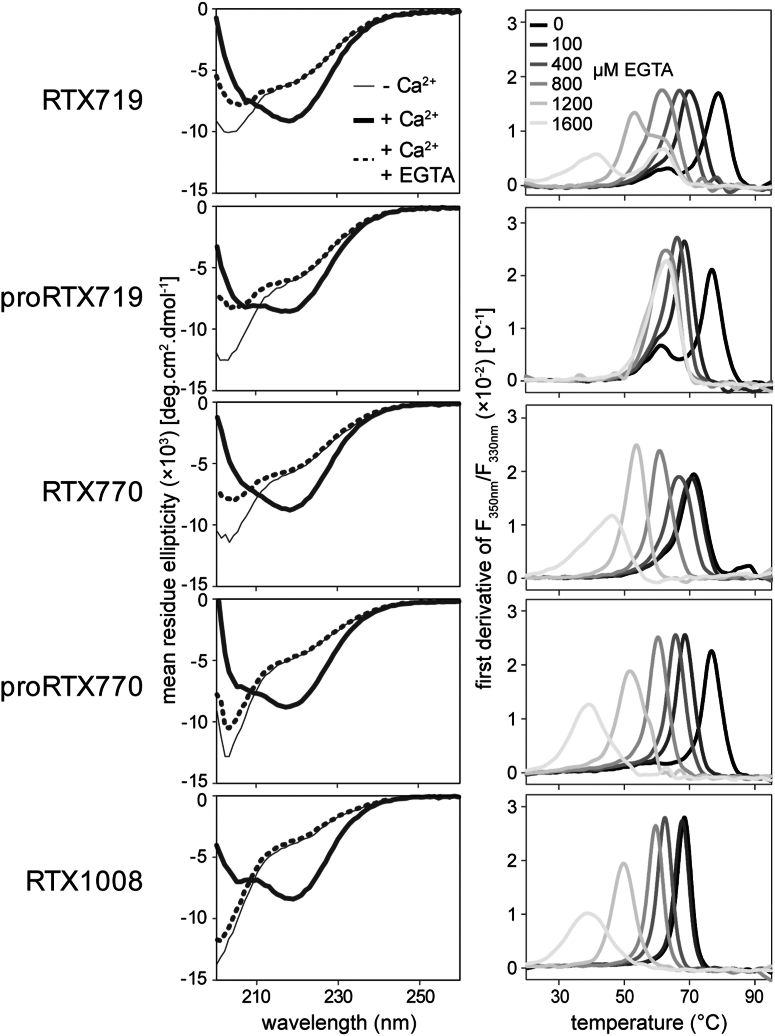
**Folding of the AD proceeds from the RTX β-roll structure**. *A*, far-UV CD spectra of RTX1008, and the acylated and nonacylated variants of the RTX770 and RTX719 constructs upon folding of the proteins in the absence (*thin line*) and the presence of 1.5 mM CaCl_2_ (*thick line*). The *dashed lines* represent the far-UV CD spectra of the Ca^2+^-loaded proteins treated with 1.6 mM EGTA. *B*, the nanoDSF thermal unfolding first derivative curves calculated as the ratio of intrinsic fluorescence intensities recorded at 350 and 330 nm (F_350 nm_/F_330 nm_). The Ca^2+^-loaded proteins were supplemented with different concentrations of EGTA (0–1.6 mM) and subjected to a linear temperature ramp in the range of 20 to 95 °C. The data are representative of two independent experiments performed in duplicate. AD, acylated domain; nanoDSF, nanoscale differential scanning fluorimetry.

To further investigate the structure-function characteristics of the AD, we prepared a series of RTX constructs in which the truncated RTX domain, capable of vectorial Ca^2+^-induced folding, was extended to residues 770 or 719 ((35) and Fig. 1). This design either included or excluded the α-helices predicted to form between residues 719 and 770, which were proposed to stabilize the AD structure (36). For this purpose, the RTX719 and RTX770 constructs were expressed in *Escherichia coli* cells in the presence or absence of the CyaC acyltransferase, yielding acylated RTX719 and RTX770 proteins, or their nonacylated proRTX719 and proRTX770 variants. The proteins were purified from 8 M urea extracts of inclusion bodies by anion exchange chromatography under denaturing conditions and were refolded by on-column size-exclusion chromatography (SEC) in the absence of calcium ions. Unlike the RTX1008 construct, which is fully unfolded in the absence of calcium (as evidenced by a prominent negative band at 200 nm in the CD spectrum), the monomeric Ca^2+^-free RTX719, proRTX719, RTX770, and proRTX770 proteins exhibited a less intense CD signal, characterized by spectra with a negative band at 203 nm (Fig. 3, left panel). This indicated a partial folding of the N-terminal segment of the AD in the absence of Ca^2+^ ions, regardless of the acylation status of the proteins. At a calcium concentration of 1.5 mM, the Ca^2+^-induced folding of the proteins yielded comparable spectra with a pronounced negative band at 218 nm indicative of a high β-sheet content. However, in contrast to the acylated RTX719 and RTX770 proteins, the spectra of the Ca^2+^-loaded proRTX719 and pro-RTX770 proteins exhibited a minor, but well-discernible negative peak at 205 nm, indicative of a slightly higher α-helical content in the nonacylated proteins. This suggested that the attached acyl chains promote local structural rearrangements during the Ca^2+^-induced protein folding.

To assess the role of Ca^2+^ ions in the stability of the AD structure, the Ca^2+^-loaded proteins were titrated with EGTA. As expected, the chelation of Ca^2+^ ions by excess EGTA (1.6 mM) led to complete unfolding of the RTX1008 polypeptide, yielding a CD spectrum like in the Ca^2+^-free buffer (Fig. 3, left panel). In contrast, the spectra of the EGTA-treated RTX770, proRTX770, RTX719, and proRTX719 proteins still exhibited a negative band at 203 nm, even though at lower intensities than the spectra of the Ca^2+^-free unfolded state. This suggested that certain structural elements in the N-terminal segment of the AD remain stable and resist 1.6 mM EGTA-induced unfolding, unlike for the RTX moiety, of which the folding/unfolding is fully reversible (37). Additionally, the EGTA-treated proteins were nearly devoid of β-sheet structures, as illustrated by the absence of the pronounced negative band at 218 nm. This highlighted an intimate structural connection between the β-rich AD and the RTX β-roll, resulting in their cooperative unfolding upon Ca^2+^ depletion.

To corroborate these observations, the stability of the Ca^2+^-loaded proteins upon titration with up to 1.6 mM EGTA was assessed through thermal unfolding experiments. The protein melting points (T_*m*_) were determined as the peak values of the first derivative of the unfolding curve, measured as the ratio of tryptophan fluorescence emission intensities at 330 and 350 nm (Fig. 3, right panel). Both the RTX770 and proRTX770 proteins exhibited monophasic curves, with the nonacylated protein showing a significantly higher T_*m*_ (80 °C) compared to the acylated variant (70 °C). In contrast, the thermal stability of the RTX719 and proRTX719 constructs was quite comparable, showing two similar T_*m*_ values (about 78 °C and 61 °C), as indicated by the biphasic nature of their melting curves. However, upon progressive Ca^2+^ chelation with increasing EGTA concentrations, the thermal unfolding curve of proRTX719 lost its biphasic character and exhibited a narrower range of T_*m*_ for the unfolding intermediates than the acylated RTX719 protein (Fig. 3, right panel). These data suggested that the N-terminal segment of the AD (residues 719–770) forms a distinct subdomain that folds independently of the acylation status and autonomously from the Ca^2+^-induced folding of the C-terminal segment of the AD.

### Fatty acyl modification modulates the folding of the AD and its stability

**Figure 4:**
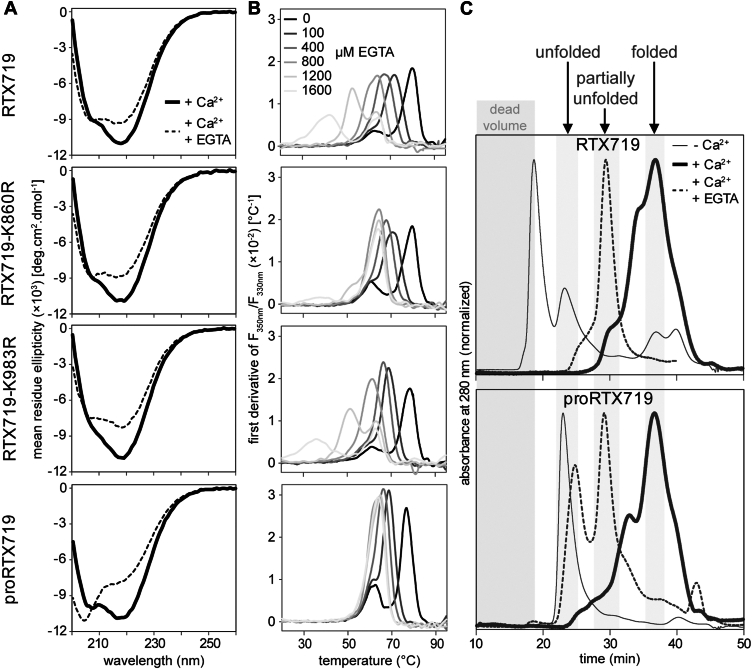
**Acyl chains affect the overall stability of the RTX719 constructs**. *A*, far-UV CD spectra of the RTX719 protein constructs upon folding in the presence of 1.5 mM CaCl_2_ (*thick line*) and unfolding in the presence of 1.6 mM EGTA (*dashed line*). *B*, the first derivative of the nanoDSF thermal unfolding curves of the RTX719 protein constructs calculated as the ratio of intrinsic fluorescence intensities recorded at 350 and 330 nm (F_350_/F_330_). The Ca^2+^-loaded proteins were supplemented with different concentrations of EGTA (0–1.6 mM) and subjected to a linear temperature ramp in the range of 20 to 95 °C. Data are representative of two independent experiments performed in duplicate. *C*, overlay of the size-exclusion chromatography elution profiles of the acylated RTX719 (*upper panel*) and the nonacylated proRTX719 (*lower panel*) proteins in the absence (*thin line*) or the presence of 1.5 mM CaCl_2_ (*thick line*). The *dashed lines* represent the chromatograms of the Ca^2+^-loaded proteins treated with 1.7 mM EGTA. nanoscale differential scanning fluorimetry.

To evaluate the individual impact of each of the two attached acyl chains on the AD structure, we individually replaced the K860 and K983 lysines with arginine residues that cannot be acylated to obtain mono-acylated RTX719-K860R and RTX719-K983R proteins. These were purified under denaturing conditions in 8 M urea and refolded by on-column refolding in the presence of 1.5 mM calcium and not by a progressive titration by Ca^2+^ ions as in the previous experiment (*c*.*f*. Fig. 3). The far-UV CD spectra of the Ca^2+^-refolded mono-acylated protein monomers were quite comparable, each displaying the characteristic negative band at 218 nm, typical of the β-rich structure of the Ca^2+^-loaded proteins (Fig. 4*A*, solid lines). In contrast, the spectrum of the Ca^2+^-refolded proRTX719 protein prepared by the same procedure exhibited a distinct negative band at 205 nm, indicating a slightly higher α-helix content in the nonacylated protein than its acylated RTX719 counterpart. The impact of acylation on the AD structure was again evident when the Ca^2+^-loaded proteins were exposed to 1.6 mM EGTA. Extraction of Ca^2+^ ions from the Ca^2+^-refolded RTX719, RTX719-K860R and RTX719-K983R proteins led to a reduction in β-sheet content, but preserved α-helical structures, as revealed by the appearance of negative bands at 205 and 222 nm (Fig. 4*A*, dashed lines). In contrast, the CD spectrum of the EGTA-treated proRTX719 exhibited a significant reduction in the negative peak at 218 nm and an increase in the intensity of the negative band at 205 nm, indicating an almost complete unfolding of the nonacylated protein upon Ca^2+^ chelation by EGTA.

Thermal unfolding experiments revealed that the thermal stability of the double-acylated, mono-acylated, and nonacylated Ca^2+^-refolded proteins was quite comparable, each showing a biphasic melting curve with transitions at approximately 78 °C and 64 °C (Fig. 4*B*). However, when exposed to increasing EGTA concentrations, the nonacylated proRTX719 exhibited a narrower range of T_*m*_ values in the presence of EGTA than the double-acylated RTX719 protein. Intriguingly, the thermal denaturation of the mono-acylated RTX719-K860R protein (acylated on K983) across increasing EGTA concentrations showed a similarly narrow range of T_*m*_ values, resembling that of the nonacylated protein. In contrast, the mono-acylated RTX719-K983R protein (acylated on K860) behaved more like the double-acylated variant, suggesting that acylation at the K860 residue plays a primary role in stabilizing the RTX719 structure upon calcium removal.

The folding status of the RTX719 and proRTX719 proteins was analyzed by assessing their hydrodynamic parameters using SEC (Fig. 4*C*). The size exclusion profile of the EGTA-treated proRTX719 revealed two peaks, eluting at 24 and 30 min. The earlier peak corresponded to the hydrodynamic radius of the fully unfolded polypeptide, while the latter represented a partially unfolded protein with a significantly larger hydrodynamic radius than the fully folded form that elutes at 36 min. In contrast, the EGTA-treated RTX719 eluted exclusively as a partially unfolded protein, indicating that the K860 and K983-linked acyl chains help to maintain the structural stability of the AD, preventing its complete unfolding despite of calcium ion removal.

### Acylation affects the compactness of the AD domain

**Figure 5:**
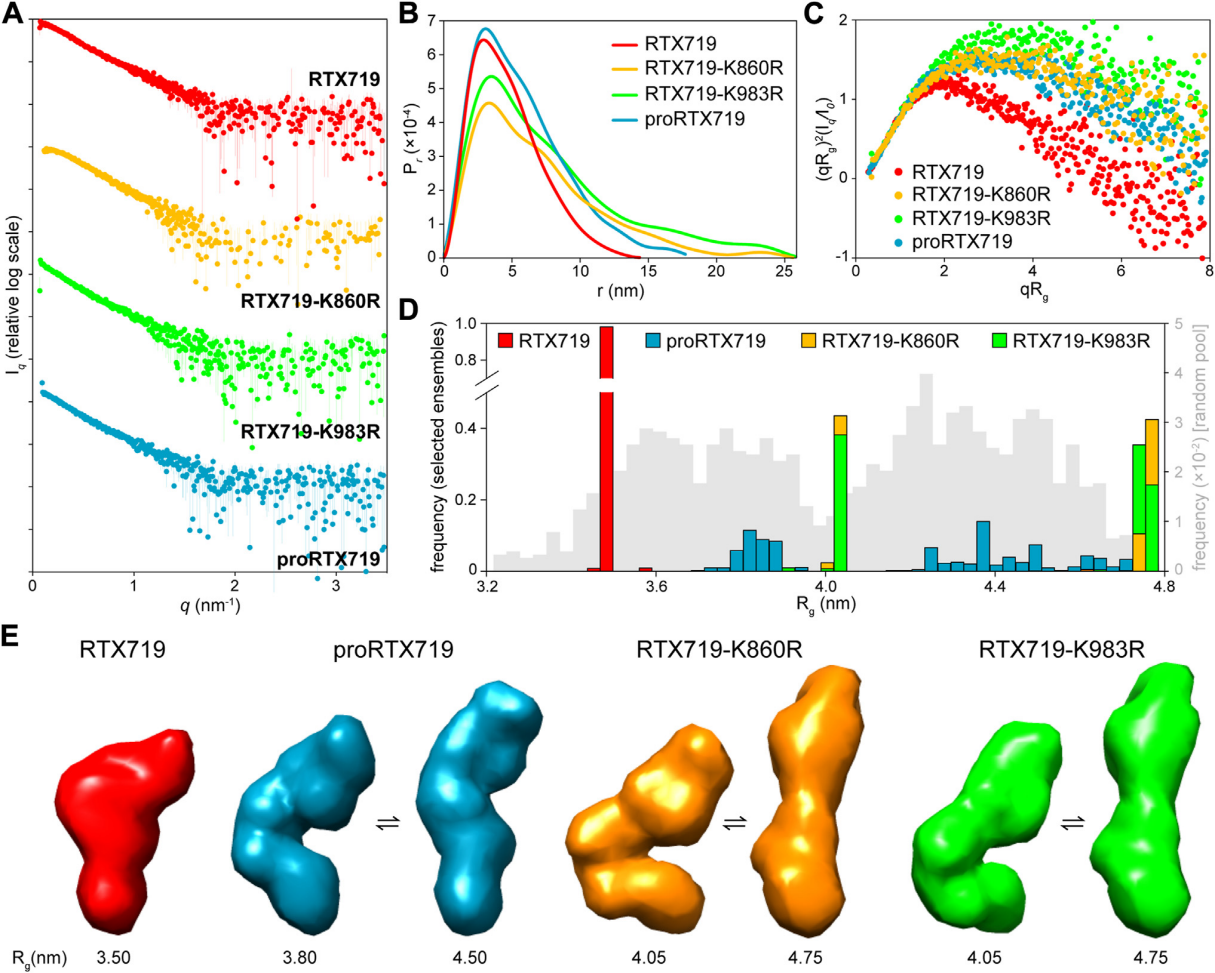
**Small-angle X-ray scattering of the Ca^2+^-loaded RTX719 protein constructs**. *A*, experimental scattering curves for RTX719 (*red*), RTX719-K860R (*yellow*), RTX719-K983R (*green*), and proRTX719 (*blue*). *B*, the pair distance distribution function, and (*C*) the normalized Kratky plot of the experimental SAXS profiles of the RTX719 protein constructs. *D*, EOM analysis of the RTX719 protein constructs. The distribution of *R*_g_ values for the initial random pool (*gray*) and the selected ensembles for RTX719 (*red*), RTX719-K860R (*yellow*), RTX719-K983R (*green*), and proRTX719 (*blue*) proteins. *E*, representative conformers of the Ca^2+^-loaded RTX719 protein constructs, as revealed by EOM analysis. Corresponding *R*_g_ values are indicated for each model. EOM, Ensemble Optimization Method.

To gain further insight into the effects of attached acyl chains on structural properties of the RTX719 protein constructs, the Ca^2+^-refolded monomers were analyzed by using an in-line SEC-SAXS analysis setup. The overall structural parameters were calculated from experimental scattering curves (Fig. 5*A*, Figs. S1-S4), and the values are summarized in Table 1. The distance distribution functions (*P*_r_), derived from scattering intensities (*I*_q_), revealed that all four protein variants adopted compact structures with an asymmetric shape (Fig. 5*B*). The maximum particle size (D_max_), estimated from the pair-distance distribution function, was larger for the mono-acylated proteins (25.8 and 26.3 nm) than for the double-acylated RTX 719 (14.5 nm) or the nonacylated proRTX719 (17.8 nm) proteins. Normalized Kratky plots revealed that the nonacylated proRTX719 and the mono-acylated protein variants were substantially less compact and/or more flexible than the double-acylated RTX719, as indicated by the significant shift of the Kratky peaks to higher *q* values and shallower decay in the mid-q region (Fig. 5*C*). The molecular masses calculated from the SAXS data were consistent with those derived from the amino acid sequences, confirming that the proteins were monomeric (Table 1). However, a nonlinearity observed in the Guinier plots for the scattering data of RTX719-K860R and RTX719-K983R indicated small amounts of protein aggregation, reducing data reliability for the mono-acylated proteins (38).

**Table 1:** Overall parameters of SAXS data

	Protein constructs			
	RTX719	RTX719-K860R	RTX719-K983R	proRTX719
SASBDB ID	SASDPP3	SASDPQ3	SASDPR3	SASDPS3
Data collection parameters				
SAXS device	SAXSpoint 2.0 (Anton Paar, Austria). Center of Molecular Structure, BIOCEV, Czech Republic			
X-ray source	MetalJet C2 (Excillum, Stockholm, Sweden)			
Beam geometry (nm)	2.0/0.983^a^			
Wavelength (nm)	0.134			
Detector	Eiger R 1M (Dectris, Switzerland)			
Detector distance (m)	0.793			
Sample cell	Flow-through quartz capillary (2 mm)			
q-range (nm^-1^)	0.069–4.298			
Exposure time per frame	15 s			
Total frames	3500	4300	3400	3500
Size-exclusion chromatography				
SEC device	Akta GO (GE Healthcare) FPLC system			
SEC column	Superdex 200 Increase 10/300			
Flow rate	0.7 ml/min, 0.01 ml/min during data acquisition			
Sample buffer solution	3 mM CaCl_2_, 50 mM NaCl, 20 mM Tris–HCl, pH 8			
Frames	1115–1528	1663–1993	2036–2289	1249–1663
Injection volume	150 μl			
Injection concentration (mg/ml)	8.513	10.831	14.264	11.788
Peak maximum concentration (mg/ml)	0.786	0.565	0.675	0.764
Structural parameters and data quality				
Data q-range (nm^-1^)	0.089–3.998	0.089–3.998	0.089–3.998	0.089–3.998
q-range from P(r) (nm^-1^)	0.16–2.34	0.14–1.6	0.094–1.51	0.11–1.9
I(0) from P(r)	0.0452 ± 0.0005	0.046 ± 0.002	0.058 ± 0.002	0.0558 ± 0.0006
I(0) from Guinier	0.0434 ± 0.0004	0.0438 ± 0.0009	0.0540 ± 0.001	0.0546 ± 0.0006
R_g_ from P(r) (nm)	3.76 ± 0.06	5.8 ± 0.5	6.6 ± 0.3	4.51 ± 0.08
R_g_ from Guinier fit (nm)	3.41 ± 0.05	5.0 ± 0.1	5.3 ± 0.1	4.2 ± 0.06
q-range for Guinier fit (nm^-1^)	0.16–0.38	0.14–0.26	0.094–0.24	0.11–0.31
Porod volume estimate (nm^3^)	67378.8	178326.0	186882.0	132616.0
Guinier aggregation index	−0.0556	0.184	0.00882	0.0198
Ambiguity score	2.246	2.809	2.34	2.17
D_max_ (nm)	14.5	25.8	26.3	17.8
Optimal Shannon channels	6.0	9.0	7.0	9.0
Optimal q_max_ (Shannon analysis)	1.73	2.03	1.9	2.25
Molecular mass determination				
From Vc	210977.0	968859.0	41613.0	73294.7
From size and shape	69004.8	120263.0	120959.0	81914.6
From Bayes	68775.0	94225.0	94225.0	83125.0
From Porod (Da)	42111.7	111454.0	116801.0	82885.0
Monoisotopic theoretical MW	78501.7	78501.7	78501.7	78501.7
Including acyl (approx.)	79051.6	78776.6	78776.6	78501.7
*Ab initio* modeling				
Number of models	32	N.D.	N.D.	32
Excluded models	2			1
Estimated molecular weight	63700.0 ± 500.0			74900.0 ± 600.0
Particle Rg (nm)	3.7577 ± 0.0003			4.5116 ± 0.0005
Particle Dmax (nm)	14.7 ± 0.3			18.2 ± 0.6
^χ2^	0.986 ± 0.001			1.156 ± 0.003
Atomistic modeling				
Model source	AlphaFold, predicted model of full-length sequence of RTX719 (October 8th, 2021)			
q-range (nm^-1^)	0.11–1.7	0.1–1.9	0.094–2.0	0.094–2.3
^χ2^	2.39	3.01	1.68	1.17
*p* value	5.59 × 10^11^	6.73 × 10^8^	2.00 × 10^6^	0.78
Flexibility analysis				
q-range	0.11–1.7	0.1–1.9	0.094–2.0	0.094–2.3
Number of input models	1500			
χ2	1.03	1.76	1.32	1.1
*p* value	0.42	2.8 × 10^-4^	1.2 × 10^-3^	0.78
*R*^flex^	2.72	28.07	29.98	73.39
*R*^random^	94.24	94.24	94.24	94.24
*R*^σ^	0.03	1.01	0.98	0.88
Ensemble *R*^g^	3.50	4.45	4.48	4.22
Ensemble D^max^	13.24	15.71	15.92	15.76
Software				
ATSAS software version	3.0.5			
Primary data reduction	Chromixs, PRIMUS			
Indirect Fourier transform	GNOM			
*Ab initio* analysis	DAMMIF/DAMMIN			
Model averaging	DAMMAVER			
Final refinement	DAMMIN			
High-resolution model	Alphafold			
Atomistic modeling	CRYSOL			
Flexibility analysis	SREFLEX, EOM 2.0			

N.D., not determined; SAXS, small angle X-ray scattering.

a Beam size at detector/beam size at sample.

The conformational flexibility of the proteins was further assessed using a combination of Normal Mode Analysis and the Ensemble Optimization Method. In this approach, a large pool of random models encompassing the conformational landscape of the AlphaFold model of the RTX719 structure was generated and a subensemble of conformers coexisting in solution were then selected based on their fit to the experimental SAXS data (Fig. 5*D*). Unlike the initial pool of models with random conformations, which displayed a broad distribution of *R*_g_ values ranging from 3.2 to 4.8 nm (gray bars), the double-acylated RTX719 protein exhibited an almost singular population of conformers with a mean *R*_g_ value of 3.5 nm (red bars). In contrast, proRTX719 showed a broader distribution of *R*_g_ values ranging from 3.7 to 4.4 nm (blue bars), indicating that the nonacylated protein adopts a more extended and flexible structure than the compact and rigid conformation of the double-acylated variant. The mono-acylated RTX719-K860R and RTX719-K983R proteins were characterized predominantly by two conformational ensembles, with *R*_g_ values of 4.0 nm and 4.75 nm (orange and yellow bars). The average ensemble *R*_g_ values aligned closely with the experimental *R*_g_ values and significantly differed from the theoretical *R*_g_ value (4.2 nm) calculated for the AlphaFold model structure. The most representative models of the protein conformers are shown in Figure 5*E*. Unlike the double-acylated RTX719 protein, which forms a compact particle with an asymmetric shape, the mono-acylated RTX719-K860R and RTX719-K983R, as well as the nonacylated proRTX719, exhibit a more elongated and highly flexible structure. Collectively, these data suggest that the presence of two fatty acyl chains induces specific conformational rearrangements that increase the structural compactness of the RTX719 structure.

### Acyl chains are sufficient for CR3-dependent anchoring of RTX719 protein into target cell membrane

**Figure 6:**
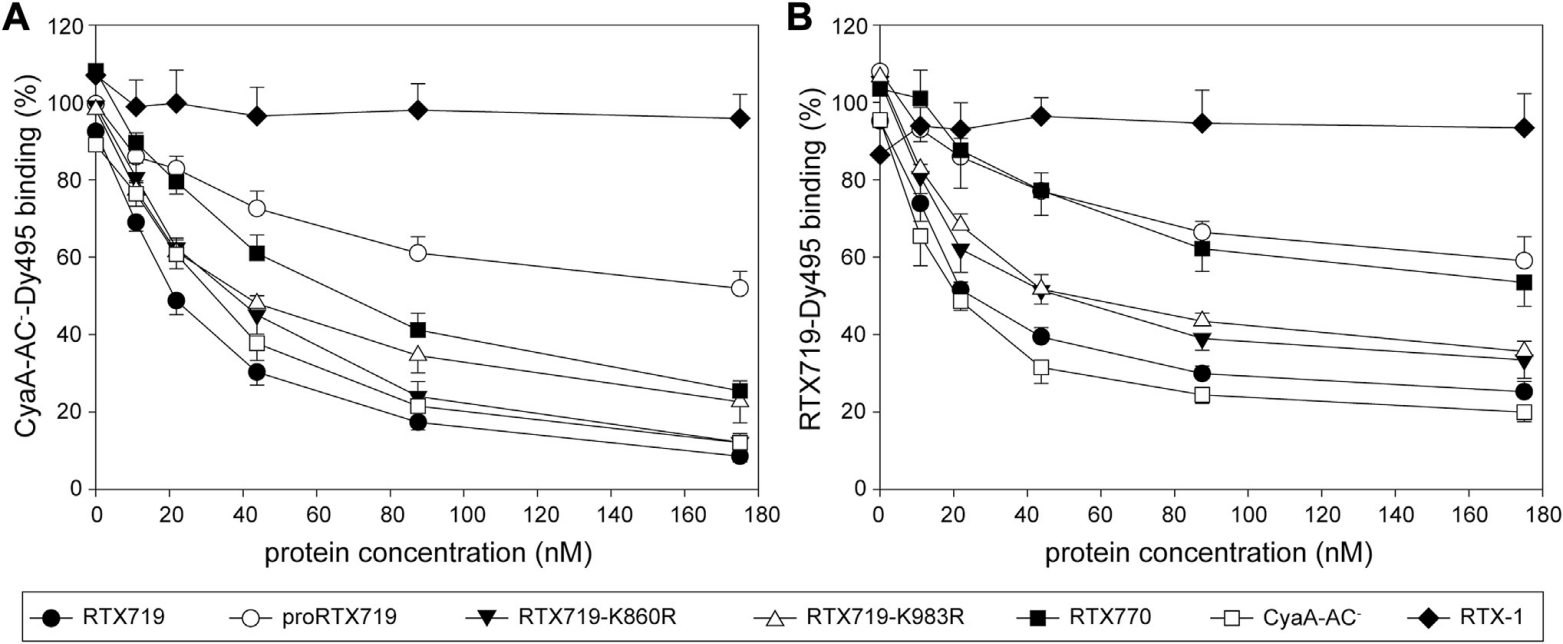
**The pore-forming domain is dispensable for the receptor-dependent anchoring of CyaA into the target cell membrane**. The CHO-CR3 cells were preincubated with the indicated concentrations of the CyaA variants for 15 min at 4 °C before supplemented with the Dy495-labeled CyaA-AC^−^ (*A*) or the Dy495-labeled RTX719 (*B*) proteins and incubated for an additional 15 min at 4 °C. The fluorescence intensity of the cell-bound Dy495-labeled proteins was then analyzed by flow cytometry. The data are expressed as the percentage of binding of the Dy495-labeled proteins, with 100% corresponding to the binding of the Dy495-labeled proteins in the absence of the competitor protein. The data represent the mean ± SD from three independent experiments. AC, adenylyl cyclase; CyaA, adenylate cyclase toxin.

We next took advantage of the absence of the transmembrane pore-forming domain of CyaA in the RTX719 construct and assessed the contribution of the attached acyl chains to CR3 receptor-mediated anchoring of the protein onto the target cell membrane. We thus examined the ability of the double-acylated, mono-acylated, and nonacylated protein constructs to compete for CR3-mediated binding to stably transfected CHO-CR3 cells using in parallel a Dyomics 495–labeled RTX719 (RTX719-Dy495) or the enzymatically inactive CyaA toxoid (CyaA-AC^-^-Dy495) as fluorescent tracers. As shown in Figure 6*A*, the previously described RTX-1 construct (35), which lacks the AD and has a low affinity for CR3 receptor binding, was fully outcompeted by the double-acylated CyaA-AC^-^-Dy495 tracer across all concentrations tested. In contrast, the double-acylated RTX719 protein, as well as its mono-acylated RTX719-K860R and RTX719-K983R variants, efficiently inhibited in a dose-dependent manner the binding of CyaA-AC^-^-Dy495 to CR3 on cell surface. This revealed that the pore-forming domain was not essential for the initial anchoring of the toxin to the membrane of CR3-expressing cells and that even a single acyl chain attached to either K860 or K983 lysine residue was sufficient for CR3-mediated interaction of the RTX719 construct with target cell membrane. This conclusion is further supported by the competitive binding curves, where the double-acylated RTX719-Dy495 performed similarly to CyaA-AC^-^-Dy495, confirming that the acylated AD alone is sufficient for CR3-mediated interaction with the target cell membrane (Fig. 6*B*). Indeed, the nonacylated proRTX719 protein competed poorly for CR3 binding with RTX719-Dy495 or with CyaA-AC^-^-Dy495. Furthermore, the double-acylated RTX719 competed more efficiently for CR3 interaction than the double acylated RTX770, indicating that the segment between residues 719 and 770 of CyaA plays a stabilizing role in the structure of the AD.

## Discussion

Using the example of the AD of the *Bordetella pertussis* CyaA, we show that this conserved and functionally important segment of the pore-forming RTX toxins plays a pivotal role in the ability of CyaA to interact with host cell membrane. The AD undergoes posttranslational acylation at specific lysine residues, which is essential for the toxin to exert its cytotoxic activities. The attached acyls enhance the hydrophobicity of the AD, anchor it onto the cell membrane surface, thereby facilitating the insertion of the membrane spanning hydrophobic domain of the toxin into the target cell membrane. In the case of CyaA this results in translocation of the AC domain into the cytoplasm of host cells and in parallel formation of oligomeric cation-selective pores in the target membrane (38, 39).

This study provides structural insight into the role of acylation in stabilizing of the Ca^2+^ binding–driven fold of the AD and reveals that the attached acyls may insert into the outer leaflet of the cell membrane and thereby stabilize the CR3-mediated association of the toxin with target cell membrane. Our folding/unfolding experiments demonstrated that the folding of the AD is closely linked to Ca^2+^-induced cooperative folding of the C terminally adjacent β-roll structure within the RTX domain. This observation is consistent with structural data, revealing that the C-terminal segment of the AD (residues 770–1000) consists of a noncanonical β-roll structure. The noncanonical β-roll is connected to the first RTX β-roll through a linker segment composed of a helical assembly of three antiparallel β-strands (6). Interestingly, the structural organization of this linker segment appears to be conserved and optimal for linking of the five β-roll structures of the RTX domain, as identical linker motifs interconnect also the other RTX repeat blocks of CyaA (31, 32). Together, the linker segment and the parallel β-shaft continue the RTX β-roll fold, constituting a contiguous β-strand assembly with a highly hydrophobic core. Given the interconnectivity of these segments and the fact that Ca^2+^-induced folding of the RTX domain proceeds successively from the C terminus toward the N terminus (24, 34), it appears that the cooperative folding of the β-roll structure propagates into and drives the folding of the AD segment. Consequently, the folding of the AD is both cooperative and templated by the adjacent β-roll, ensuring proper structural arrangement of the AD. The importance of proper arrangement of the RTX β-roll for successful β-roll formation in the AD is highlighted by the differences in the secondary structure content observed between the AD-RTXa and AD-RTXb proteins upon calcium binding (*c*.*f*. Fig. 2). Misalignment of the RTX motifs resulted in an out-of-frame folding of the β-roll, leading to a complete misfolding of the AD polypeptide. Similarly, the removal of the aromatic ring of Y940, located in the position of a strictly conserved core-facing tyrosine in the linker segment, has been shown to induce misfolding and aggregation of the toxin (40). Thus, the cooperativity of the folding process appears to be crucial for the maintenance of a functional conformation of the AD segment.

Our data further demonstrated that while the C-terminal portion of the AD (residues 770–1000) is structurally linked to the RTX β-roll, the N-terminal segment of the AD (residues 719–770) exhibits some degree of structural independence. This is particularly evident from the thermal stability experiments and EGTA-induced unfolding assays, showing that the N-terminal region of the AD retains some structural stability even upon the extraction of Ca^2+^ ions from the structure by excess EGTA (*c*.*f*. Fig. 3). In fact, a *de novo* protein structure prediction by AlphaFold suggests that the N-terminal segment of the RTX719 polypeptide forms a bundle of three α-helices, positioned adjacent to the tips of the long loops carrying the acyls on the ε-amino groups of residues K860 and K983. Moreover, a structural similarity search indicated that the three-helix bundle would resemble the fold of an ACP. Indeed, the ACPs are small, highly conserved proteins consisting of an α-helical bundle structure and serve to traffic acyl chains in the fatty acid biosynthesis pathway (41). Furthermore, acyl-ACPs also serve as fatty acyl donors for the highly selective acylation of RTX toxins by dedicated acyltransferases, such as CyaC (42). In this process, a fatty acyl chain is attached to the ACP *via* the 4’-phosphopantetheine prosthetic group and positioned into a hydrophobic surface groove (43). Hence, the proximity of the three-helix bundle to the acylation sites in the CyaA structure, along with its structural resemblance to ACPs, could imply that the N-terminal segment of the AD forms a similar hydrophobic groove, which can accommodate the acyl chain protruding from the K860 or K983 residue (or both). This groove could then serve to shield the acyl chain from solvent exposure, ensuring that the AD maintains its functional conformation throughout the folding process.

Posttranslational acylation of the K860 and K983 residues of CyaA has been repeatedly shown to be critical for both the pore-forming activity of the toxin and for its capacity to intoxicate host cells by translocating the AC enzyme domain into cells (25, 44). However, the acyl chains themselves are not required for the Ca^2+^ binding–riggered folding of the AD and RTX domain, as evidenced by comparable structural characteristics of the acylated and nonacylated RTX719 constructs (*c*.*f*. Fig. 4). Indeed, the 2.7 Å cryo-EM map of the Ca^2+^-loaded RTX751 construct (residues 751–1706 of CyaA) was derived from the nonacylated polypeptide (6). The SAXS-derived structural models revealed that the double-acylated RTX719 protein adopts a more compact, “closed” conformation, whereas the mono-acylated and nonacylated proteins exhibit greater flexibility and favor an “extended” polypeptide conformation (*c*.*f*. Fig. 5*E*). A similar extended conformation is observed in the RTX751 structure derived from a nonacylated polypeptide (6). This structure provides a structural basis for the interaction of CyaA with the heterodimeric CR3 integrin cell surface receptor and likely templates the AlphaFold model of nonacylated proRTX719. However, alignment of the integrin-binding site from the double-acylated RTX719 with that of nonacylated proRTX719 within the RTX751–CR3 complex revealed that the closed conformation of the double-acylated protein fails to dock *in silico* with the CR3 receptor due to steric clashes (Fig. S5). Nevertheless, the double-acylated RTX719 protein fully outcompeted the full-length toxin for CR3 binding on the cell surface, suggesting that the closed conformation retains the capacity of RTX719 to engage CR3 *in vitro* (*c*.*f*. Fig. 6). This discrepancy may be explained by the fact that both CyaA and CR3 are structurally flexible and undergo substantial conformational rearrangements to carry out their biological activities. Indeed, depending on activation state, CR3 cycles between open and bent conformations (45), while CyaA is thought to insert its hydrophobic domain into the lipid bilayer and translocate its N-terminal AC domain into the host cell cytosol (8, 46). Therefore, a single static representation of the CyaA–CR3 interaction is likely inadequate to capture the full complexity of this dynamic process.

We further demonstrated that the presence of the acyl chains in the double-acylated protein significantly enhances the structural stability of the AD. This was evident from the unfolding experiments, in which the nonacylated, Ca^2+^-loaded proRTX719 protein was more susceptible to the EGTA-induced unfolding than its acylated, Ca^2+^-loaded counterparts (Fig. 4). These findings are consistent in with the previous report showing that acylation significantly stabilizes both the hydrophobic domain and the acylated region of CyaA (47). In fact, the binding of a fatty acyl to ACP has been shown to shift the conformational equilibrium from a dynamically disordered form to the folded state of acyl-ACP, highlighting the key role of acylation in stabilizing protein structures (43). Based on these observations, we propose that the Ca^2+^-induced folding of the C-terminal segment of the AD cooperates with acylation-driven conformational rearrangements within the N-terminal AD segment. Specifically, an α-helical bundle in the N-terminal AD segment, which folds independently of its C-terminal part in the absence of calcium, would form fatty acyl binding sites that shield the acyl chains linked to the ε-amino groups of lysine residues K860 and K983, located at the tip of the long loops protruding from the C-terminal AD segment (6). This recruitment of the acyl chains would promote local conformational changes in the N-terminal AD segment, which would subsequently be transmitted back to the C-terminal AD segment, ultimately resulting in a significant increase in the stability of its structure. Thus, the presence of the acyl chains in the CyaA structure would not only be essential for its membrane-targeting activity but would also play a critical role in the folding and functional integrity of the toxin protein structure as such. This would also suggest why the biological activity of CyaA strictly depends on the modification of its K983 lysine residue by 16-carbon acyl chains and attachment of a shorter acyl does not match the structure-function requirements and fails to confer a membrane-penetrating capacity on CyaA (27, 48, 49).

From a functional perspective, the acyl modifications of the K860 and K983 residues of the AD appear to play a key role in the receptor-mediated interaction with the plasma membrane of target cells (4). One key finding of this study is that acylation of either the K860 or the K983 lysine was sufficient for tight interaction of the RTX719 construct with the toxin receptor CR3 and with the cell membrane. This goes well with the previous observation of Masin and colleagues who used the full-length CyaA molecule in CR3 binding experiments (28). This is demonstrated by the competitive binding assays, where the double-acylated RTX719 was able to fully outcompete intact CyaA from CR3 binding despite lacking the membrane-penetrating pore-forming domain (Fig. 6). Similarly, both mono-acylated variants RTX719-K860R and RTX719-K983R competed comparably well as the double-acylated RTX719 for CR3 binding, suggesting that single acylation is sufficient for anchoring of the toxin to the outer leaflet of lipid bilayer of the cell membrane. Indeed, a partial redundancy of the roles of the K860 and K983-attached acyls in CR3-mediated cell binding activity of CyaA has been previously observed (28). The poor capacity of the nonacylated proRTX719 construct to compete for CR3 binding with the acylated protein variants further highlights the critical role of the attached acyl chains in anchoring of the toxin molecule to the target cell membrane, a process that would occur independently and prior to the insertion of the pore-forming domain of CyaA into cell membrane.

In conclusion, our findings demonstrate that acylation of the K860 and K983 residues is essential for stabilizing of the AD structure and for CR3-mediated insertion of the toxin into the plasma membrane of target cells. During the Ca^2+^-driven assembly of the CyaA structure, the acyl chains, protruding from the tip of the long loops within the C-terminal AD segment would interact with the N-terminal segment of the AD, containing putative acyl binding site(s) arranged in an α-helical structure independent structurally of the C-terminal β-rich AD segment. It is plausible to speculate that the AD would function as a “folding interface,” forming a structural bridge between the N- and C-terminal parts of the large CyaA molecule. The C-terminal β-rich structures of the RTX domain, formed *via* vectorial Ca^2+^-driven folding, support efficient secretion of the toxin from bacterial cells (24) and mediate the targeting of the plasma membrane of host cells through interaction with the CR3 receptor (5, 6). In turn, the N-terminal portion of the toxin may fold independently of Ca^2+^ ions, adopting predominantly α-helical structures essential for membrane insertion of the hydrophobic pore-forming domain, translocation of the N-terminal AC domain across cell membrane and the parallel formation of cation-selective pores (30, 33, 50).

## Experimental procedures

### Cells and growth conditions

Chinese hamster ovary cells expressing human CD11b/CD18 (CHO-CR3) were cultured at 37 °C under a humidified 5% CO_2_ atmosphere in F12 medium (Gibco Invitrogen) supplemented with 10% (v/v) fetal calf serum (Sigma) and antibiotic/antimycotic solution (Sigma). The *E*. *coli* XL1-Blue (Stratagene) and BL21λ(DE3) cells transformed with the appropriate vectors were grown in Luria–Bertani medium or on Luria–Bertani agar plates in the presence of kanamycin (60 μg/ml) or ampicillin (150 μg/ml).

### Plasmid preparation

The pET28b-AD-RTXa and pET28b-AD-RTXb vectors, encoding the AD-RTXa and AD-RTXb proteins were prepared by PCR mutagenesis. The nucleotide sequence of the *cyaA* gene encoding the residues 881 to 1038 (for AD-RTXa) and 881 to 1047 (for AD-RTXb) and 1562 to 1681 was amplified by PCR using pairs of primers (Table 2) and cloned into the NcoI/HindIII-digested pET28b vector using a Gibson assembly protocol. The expression vector encoding the RTX719 protein was derived from pT7CT7ACT1-ΔNdeI, a bicistronic vector encoding the structural *cyaA* gene and the *cyaC* gene for the dedicated acyltransferase (51). For construction of the pT7CT7-RTX719 plasmids, the PCR fragments amplified from pT7CT7ACT1-ΔNdeI using a pair of primers (719For, 719Rev) was digested with NdeI/SacI and ligated together with the 782-bp SacI/AccIII fragment of pET42b-TEV-RTX-1 (35) into the NdeI/AccIII-cleaved pT7CT7ACT1-ΔNdeI vector. The expression vector encoding the nonacylated RTX719 protein (pT7Cstop-RTX719) was prepared by disruption of the *cyaC* gene in the pT7CT7-RTX719 plasmid by using ligation of the self-annealing oligonucleotide 5′-TAATCGATTAGGCC-3′ into the ClaI-linearized pT7CT7-RTX719 plasmid. The expression vectors encoding the mono-acylated variants of the RTX719 protein (pT7CT7-RTX719-K860R and pT7CT7-RTX719-K983R) were generated by exchange of the EcoRV-XhoI fragment of the pT7CT7-RTX719 plasmid (535 bp) by the 535-bp EcoRV-XhoI fragment of the pT7CACT1-K860 R and pT7CACT1-K983R vectors previously prepared (28, 52).

**Table 2:** List of primers

Primer	Sequence (5′ – 3′)
CyaA881-F1	AACTTTAAGAAGGAGATATACCATGGGCAGCAGCCATCATCATCATCATCACGGCGCGGCCGACACCAC
CyaA1038-R1	CCCTCGTCGCCCAGCAGGACGTCGTCGCCGGATCCGCCGG
CyaA1038-F2	CCGGCGGATCCGGCGACGACGTCCTGCTGGGCGACGAGGG
CyaA1047-R1	ATGGCGGCGCCGGCAACGACGTCCTGCTGGGCGACGAGGG
CyaA1047-F2	CCCTCGTCGCCCAGCAGGACGTCGTTGCCGGCGCCGCCAT
CyaA1681-R2	TCGAGTGCGGCCGCAAGCTTTTAGGGGTCCGGAT
719-For	ATACATATGCATCATCATCATCATCATGAAAAGCTGGCCAACGATTAC
719-Rev	CCAGAGCTCGTTGTCCTGG

### Protein production and purification

The proteins were produced in *E*. *coli* BL21/pMM100 cells grown at 37 °C in medium containing 95 mM Na_2_HPO_4_, 22 mM KH_2_PO_4_, 85 mM NaCl, 20 g/l of yeast extract and 20 g/l of glycerol supplemented with appropriate antibiotic. Induction of the bacterial cells was achieved using 0.5 mM IPTG at an optical density at 600 nm of ∼0.6. After 4 h, the bacterial cells were collected by centrifugation at 4,000*g* for 20 min at 4 °C, washed with TN buffer (50 mM Tris–HCl, pH 8.0, and 150 mM NaCl) and disrupted by sonication (Misonix). Unbroken cells were removed by centrifugation at 4,000*g* for 10 min at 4 °C and the supernatant was centrifuged at 30,000*g* for 30 min at 4 °C. The pelleted inclusion bodies were resuspended in TU buffer (50 mM Tris–HCl, pH 8.0, 8 M urea), and the urea extracts were clarified by centrifugation at 30,000*g* for 30 min at 4 °C.

For purification of the AD-RTXa and AD-RTXb proteins, the clarified urea extracts were loaded on a Ni Sepharose 6 Fast Flow column (Cytiva), pre-equilibrated with TU buffer. The purified proteins were eluted from the column with TU buffer supplemented with 200 mM imidazole after extensive washing of the column with TU buffer containing 25 mM imidazole. The urea-denatured proteins were then refolded on a Superdex 200 Increase 10/300 column (Cytiva) equilibrated with TN buffer. Collected fractions were concentrated by ultrafiltration using Amicon YM10 membrane (Millipore) and stored at −20 °C.

For purification of the acylated and nonacylated variants of RTX719, RTX770, and RTX1008 proteins, the clarified urea extracts were loaded onto a DEAE-Sepharose column (Sigma), equilibrated with TU buffer containing 50 mM NaCl (TUN buffer). The column was washed with TUN buffer supplemented with 1% Triton X-114 to remove *E*. *coli* lipopolysaccharide, and the detergent removal was carried out by extensive washing with TU buffer supplemented with 25 mM NaCl. The purified proteins were then eluted from the column with TU buffer containing 300 mM NaCl. The protein fractions were concentrated by ultrafiltration using Amicon YM10 membrane (Millipore) and stored at −20 °C. The RTX-1 protein was purified as described previously (35). Native proteins in monomeric state were obtained after injection of 500 μl of urea-denatured proteins at a concentration of 5 mg/ml on a Superdex 200 HR 10/300 column (GE Healthcare), equilibrated with buffer containing 20 mM Tris–HCl (pH 8.0) and 50 mM NaCl or 20 mM Tris–HCl (pH 8.0), 50 mM NaCl and 1.5 mM CaCl_2_ for Ca^2+^-free and Ca^2+^-loaded proteins, respectively. The purity and stability of the proteins was monitored by SDS-PAGE. Protein concentrations were determined spectrophotometrically using a DS-11 Fx microvolume spectrophotometer (Denovix) or by Bradford assay (Bio-Rad) using bovine serum albumin as a standard.

### Circular dichroism

The far-UV spectra were recorded at 25 °C on a Chirascan-plus spectrometer (Applied Photophysics) with a scanning speed of 1 nm/s, integration time of 1 s and at least two accumulations, in the range of 200 to 260 nm using a 0.1 cm pathlength cell (quartz Suprasil 1110-QS, Hellma). For folding studies, incremental concentrations of CaCl_2_ were added to the Ca^2+^-free proteins diluted to a final concentration of 0.2 mg/ml in 20 mM Tris–HCl (pH 8.0) and 50 mM NaCl. For the unfolding studies, the Ca^2+^-loaded proteins were supplemented with increasing concentrations of EGTA (0–1.6 mM). Spectra of the buffers were subtracted from the protein spectra and converted from degrees of ellipticity (mdeg) to mean residue ellipticity (deg·cm^2^·dmol^-1^).

### Thermal stability

Thermal stability assays were performed by nanoscale differential scanning fluorimetry using a Prometheus NT.48 device (NanoTemper Technologies). The Ca^2+^-loaded protein samples (0.1 mg/ml) were supplemented with increasing concentrations of EGTA (0–1.6 mM), incubated at 25 °C for 30 min, and loaded into nanoscale differential scanning fluorimetry grade standard capillaries (NanoTemper Technologies). The measurements were conducted from 20 to 95 °C (with a temperature ramp of 2 °C/min) under constant monitoring of tryptophan fluorescence at 350 and 330 nm. The melting temperature (*Tm*) values, corresponding to the inflection points of the unfolding curve, were determined by using PR.ThermControl software (https://nanotempertech.com/prometheus/nt48-software) (NanoTemper Technologies).

### Small angle X-ray scattering

The SAXS data were collected at the Centre of Molecular Structure of the Institute of Biotechnology of the Czech Academy of Sciences in Vestec on a SAXSpoint 2.0 instrument (Anton Paar), equipped with a MetalJet C2 X-ray source (Excillum) and an Eiger R 1M detector (Dectris) at a sample-detector distance of 0.8 m and at a wavelength of λ = 0.134 nm. To separate the monomeric forms of the proteins, SAXS data were collected on solutions eluting on-line from a Superdex 200 Increase 10/300 column (GE Healthcare) connected to an Akta GO chromatography system (GE Healthcare). The proteins were injected onto the column equilibrated with 20 mM Tris–HCl (pH 8.0), 50 mM NaCl and 3 mM CaCl_2_, and run through flow-through quartz capillary (2 mm in diameter). The separation flow rate was set to 0.7 ml/min and reduced to 0.01 ml/min when the peak appeared in the chromatogram. The protein concentrations were estimated from UV absorption at 280 nm using a Cary 60 spectrometer (Agilent Technologies) at the same place as SAXS. The SAXS signal was continuously measured with exposure time of 15 s per frame. The scattering data were averaged and normalized to the intensity of the incident beam (measured using semitransparent beam stop) and analyzed using Chromixs (53). Sample (peak) and buffer region were individually averaged and scaled to common noise level before buffer subtraction in Primus (54). The data processing and *ab initio* modeling were performed using ATSAS 3.0 software package (https://www.embl-hamburg.de/biosaxs/download.html) (55). The flexibility analysis was assessed by using a combination of normal mode analysis in SREFLEX (56) and the Ensemble Optimization Method (57). Here, five models were obtained from AlphaFold and automatically segmented into three domains (residues 1–70, 71–417, and 418–728) based on protein dynamics predicted by normal mode analysis in SREFLEX. Subsequently, 300 putative conformers were generated for each model using SREFLEX in pool mode, resulting in a total pool of 1,500 conformers. This pool was then analyzed and compared to experimental data using a Genetic Algorithm Judging Optimisation of Ensembles from the Ensemble Optimization Method.

### Analytical size-exclusion chromatography

The analytical runs of size-exclusion chromatography were carried out using a Superdex 200 Increase 10/300 GL column (GE Healthcare) connected to an AKTA pure liquid chromatography system (GE Healthcare). Size exclusion profiles of Ca^2+^-free proteins were obtained by injecting 500 μl of Ca^2+^-free proteins at a concentration of 0.5 mg/ml, which were acquired as fractions after on-column refolding of urea-denatured proteins in the buffer containing 50 mM Tris–HCl (pH 8.0) and 150 mM NaCl. Size exclusion profiles of Ca^2+^-loaded proteins were obtained by injecting 500 μl of urea-denatured proteins (0.5 mg/ml) supplemented with 1.5 mM CaCl_2_ on the column equilibrated with the buffer containing 50 mM Tris–HCl (pH 8.0), 150 mM NaCl, and 2 mM CaCl_2_. Size exclusion profiles of Ca^2+^-depleted proteins were obtained by injecting 500 μl of Ca^2+^-loaded proteins (0.5 mg/ml) on the column equilibrated with 50 mM Tris–HCl (pH 8.0), 150 mM NaCl, and 1.7 mM EGTA. All runs were performed at a flow rate of 0.5 ml/min, and protein elution was monitored by absorbance at 280 nm.

### Competition assay on CHO-CR3 cells

Competitive binding of the proteins in the presence of Dy495-labeled CyaA-AC^−^ or Dy495-labeled RTX719 was determined by flow cytometry as previously described (35). In brief, aliquots of CHO-CR3 cells (1 × 10^5^) were incubated on ice for 15 min in 200 μl of buffer containing 10 mM Hepes, pH 7.4, 140 mM NaCl, 5 mM KCl, 2 mM CaCl_2_, 2 mM MgCl_2_, 1% (w/v) glucose, and 1% (v/v) fetal calf serum in the presence of the tested competitor proteins at concentrations of 0 to 175 nM. Next, the cells were incubated with Dy495-labeled CyaA-AC^−^ or Dy495-labeled RTX719 (5.6 nM) for 30 min on ice and analyzed by flow cytometry (FACS LSR II, BD Biosciences) in the presence of 1 μg/ml of Hoechst 33258 vital dye. Data were analyzed using the FlowJo software (https://www.flowjo.com) (Tree Star) and appropriate gating was used to exclude cell aggregates and dead cells. Binding data were deduced from the mean fluorescence intensities of the cell-associated fluorescent proteins, where mean fluorescence intensities of the cell-bound Dy495-labeled proteins in the absence of competitor protein were taken as 100%.

## Data availability

The SAXS data and models of the acylated, mono-acylated, and non-acylated variants of the RTX719 protein are deposited in the Small Angle Scattering Biological Data Bank (http://www.sasbdb.org) under accession codes SASDPP3, SASDPQ3, SASDPR3, and SASDPS3, respectively. Raw SAXS data are deposited in Zenodo and available at https://doi.org/10.5281/zenodo.14651652, https://doi.org/10.5281/zenodo.14651825, https://doi.org/10.5281/zenodo.14671977, and https://doi.org/10.5281/zenodo.14672110.

## Supporting information

This article contains supporting information.

## Conflict of interest

The authors declare that they have no conflicts of interest with the contents of this article.
