# Supplementary material for: Acyl chains stabilize the acylated domain and determine the receptor-mediated interaction of the Bordetella adenylate cyclase toxin with cell membrane
Source: J Biol Chem. 2025 Jun 19;301(7):110392. doi: 10.1016/j.jbc.2025.110392 (PMC12332401; doi:10.1016/j.jbc.2025.110392)
Supplement: Supporting Material [file mmc1.pdf]

Supporting Information

Acyl chains stabilize the acylated domain and determine the receptor-mediated interaction of the *Bordetella* adenylate cyclase toxin with cell membrane

Carlos Espinosa-Vinals[1], Jan Stransky[2], Radim Osicka[1], Adriana Osickova[1], David Jurnecka[1], Peter Sebo[1], Ladislav Bumba[1*]

[1]Institute of Microbiology of the Czech Academy of Sciences, Videnska 1083, 142 20 Prague, Czech Republic.
[2]Institute of Biotechnology of the Czech Academy of Sciences, Prumyslova 595, 252 50 Vestec, Czech Republic.

[*] Correspondence: Ladislav Bumba, Institute of Microbiology of the Czech Academy of Sciences, Videnska 1083, 142 20 Prague 4, Czech Republic.
E-mail: bumba@biomed.cas.cz

Figure S1.

SAXS analysis of RTX719. A – Raw data (reduced and buffer subtracted); B – Guinier plot; C – Kratky plot; D, E – Inverse Fourier transformation (GNOM); F – Fit of AlphaFold model (Crysol); G – Fit of Ensemble optimization method (GAJOE); H – SEC-SAXS chromatography: average intensity for selected q-range and absorption spectra of selected wavelength as function of SAXS frame and wall time, frames 1115-1528 used for analysis.

Figure S2.

SAXS analysis of RTX719-K860R. A – Raw data (reduced and buffer subtracted); B – Guinier plot; C – Kratky plot; D, E – Inverse Fourier transformation (GNOM); F – Fit of AlphaFold model (Crysol); G – Fit of Ensemble optimization method (GAJOE); H – SEC-SAXS chromatography: average intensity for selected q-range and absorption spectra of selected wavelength as function of SAXS frame and wall time, frames 1663 - 1993 used for analysis.

Figure S3. SAXS analysis of RTX719-K983R.

A – Raw data (reduced and buffer subtracted); B – Guinier plot; C – Kratky plot; D, E – Inverse Fourier transformation (GNOM); F – Fit of AlphaFold model (Crysol); G – Fit of Ensemble optimization method (GAJOE); H – SEC-SAXS chromatography: average intensity for selected q-range and absorption spectra of selected wavelength as function of SAXS frame and wall time, frames 2036 - 2289 used for analysis.

Figure S4.

SAXS analysis of proRTX719. A – Raw data (reduced and buffer subtracted); B – Guinier plot; C – Kratky plot; D, E – Inverse Fourier transformation (GNOM); F – Fit of AlphaFold model (Crysol); G – Fit of Ensemble optimization method (GAJOE); H – SEC-SAXS chromatography: average intensity for selected q-range and absorption spectra of selected wavelength as function of SAXS frame and wall time, frames 1249 - 1663 used for analysis.

Figure S5.

Alignment of the AlphaFold model (upper right), and the putative model of the double-acylated (bottom left) and the non-acylated (bottom right) RTX719 constructs onto the RTX751 structure in complex with the ectodomain of the heterodimeric CR3 integrin receptor (upper left). All models were aligned based on the Cα atoms of the integrin-binding site (residues 1166–1281 of CyaA). The RTX719 polypeptide is shown in rainbow coloring, with

the N-terminus in blue and the C-terminus in red. The heterodimeric CR3 integrin receptor is depicted as a surface representation, with the α_M (CD11b) and β_2 (CD18) subunits shown in light and dark grey, respectively. Calcium ions are shown as magenta spheres. The locations of the acylation sites at residues K860 and K983 are indicated.

Figure S1

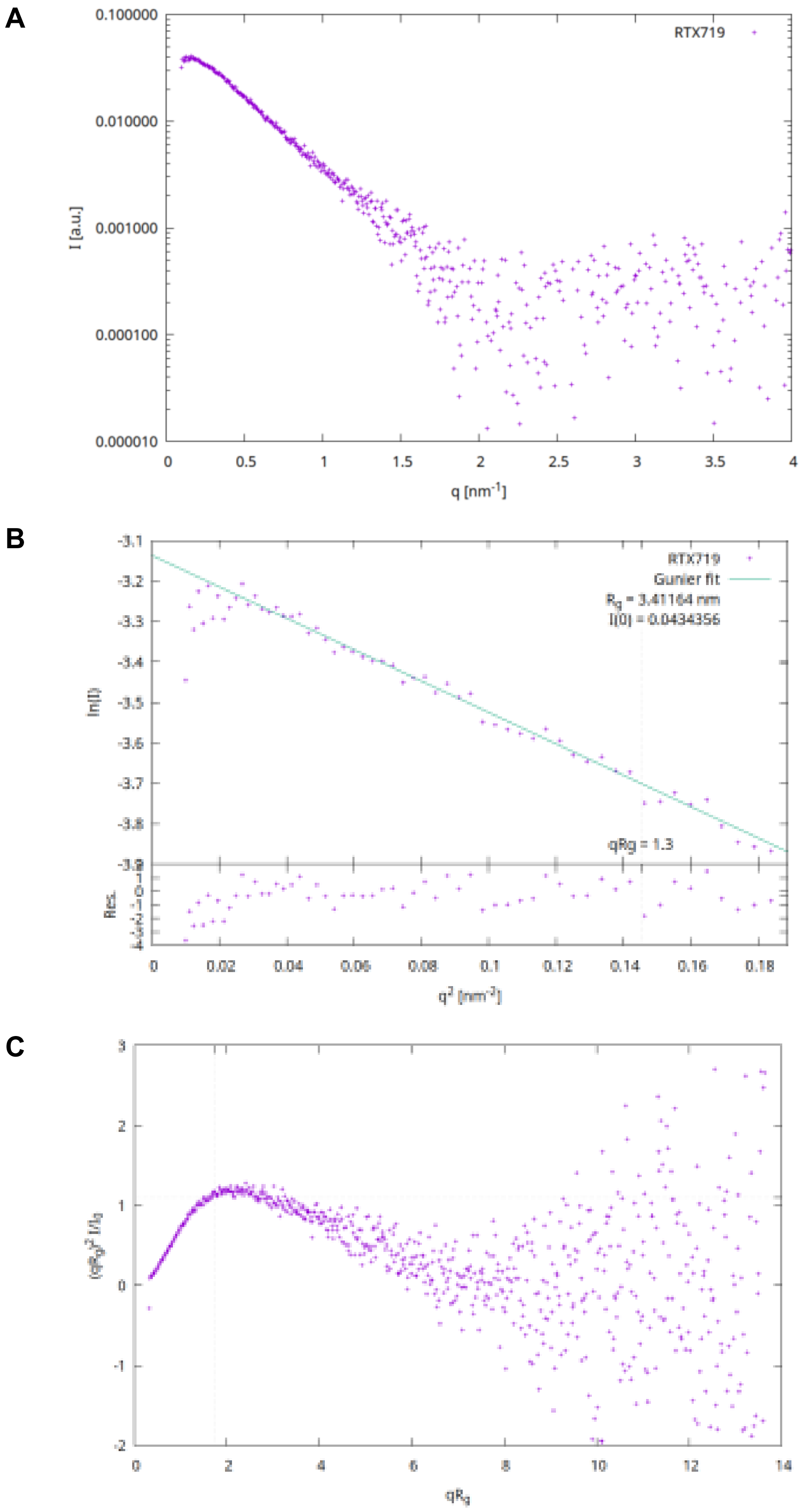
A
B
C
RTX719
I [a.u.]
q [nm^{-1}]
0.100000
0.010000
0.001000
0.000100
0.000010
Gunier fit
R_g = 3.41164 nm
I(0) = 0.0434356
qRg = 1.3
ln(I)
Res.
q^2 (nm^{-2})
$(qR_g)^2$ I/I_0
qR_g

Figure S1 (continued)

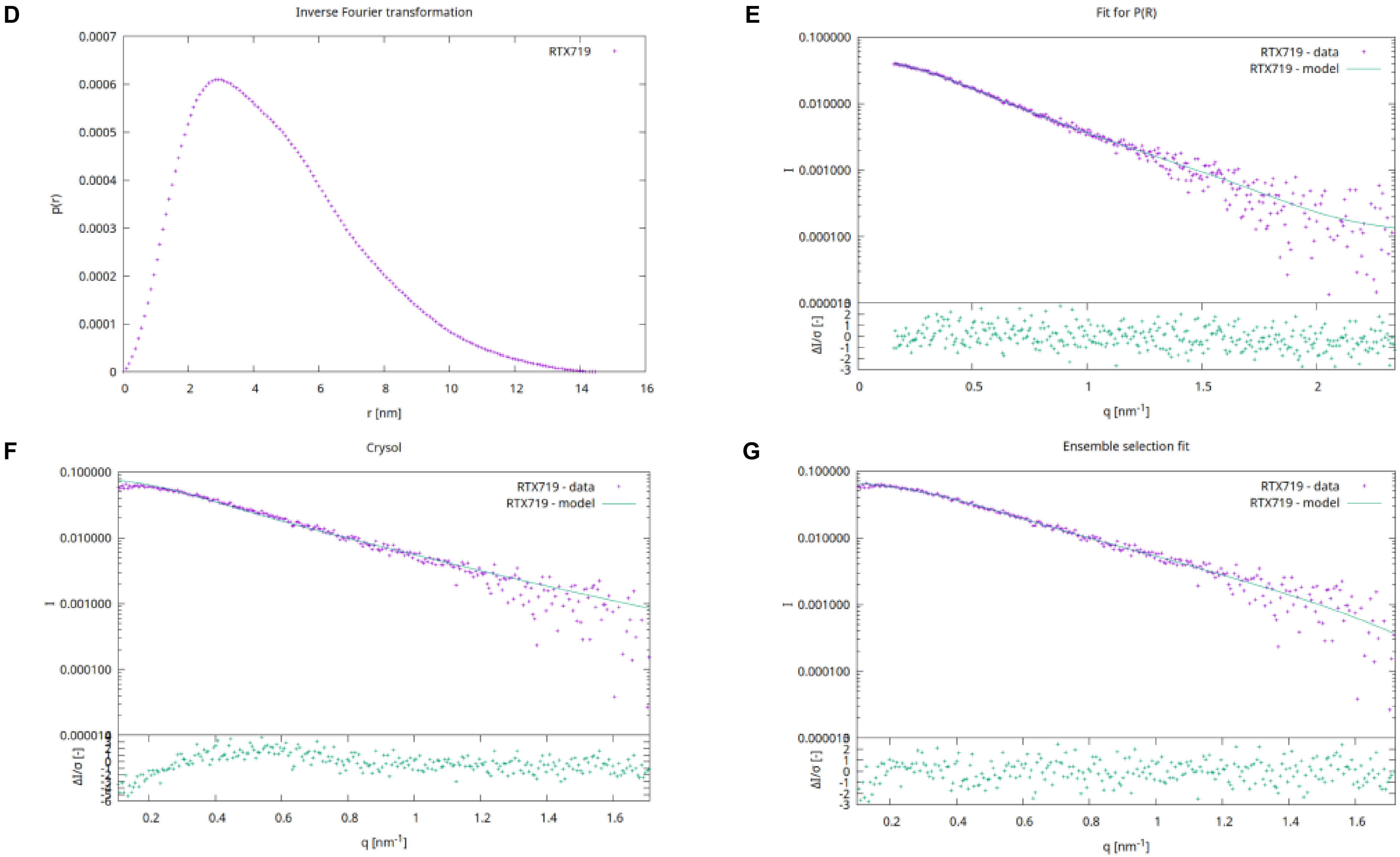

Figure S1 (continued)

H

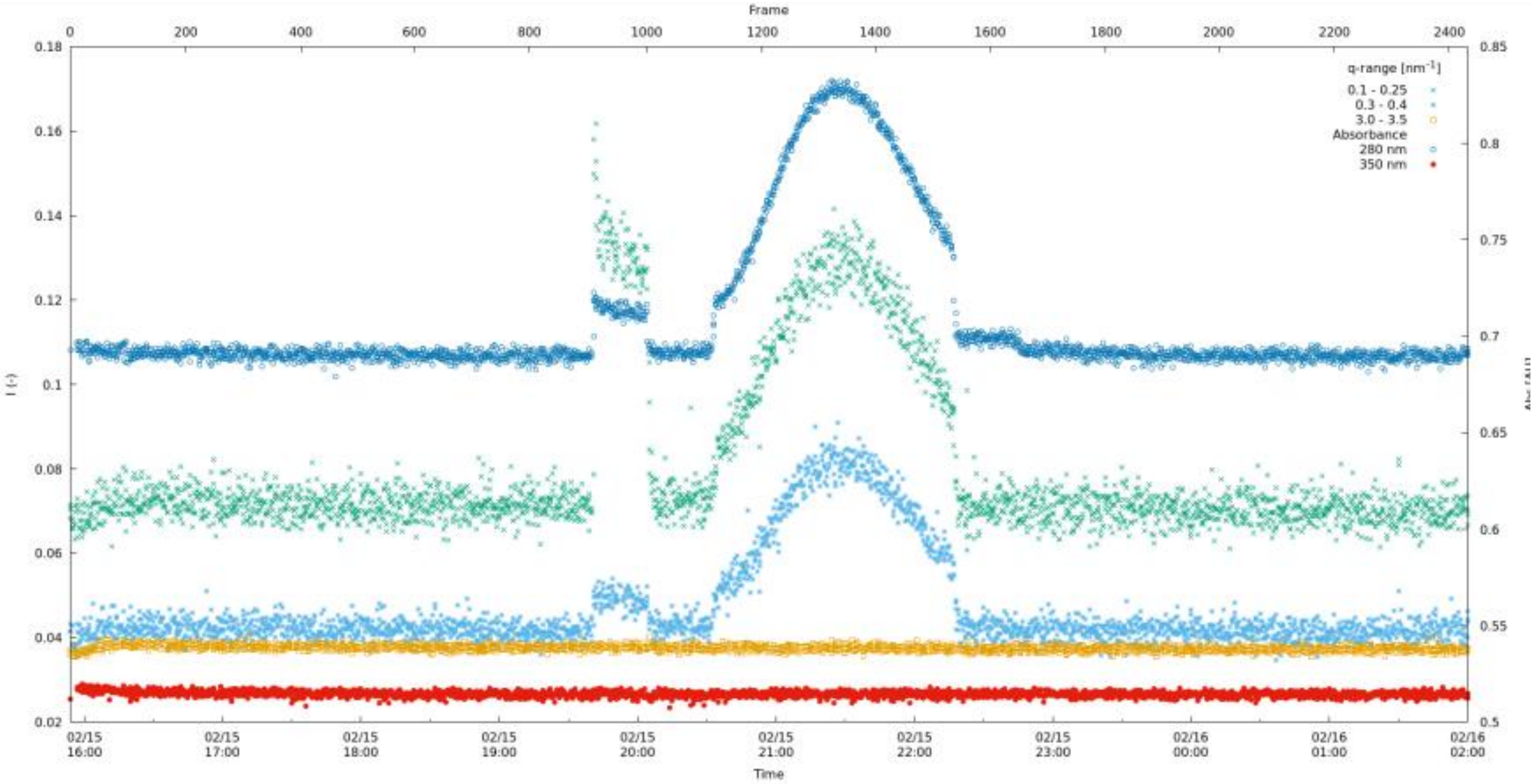

Figure S2

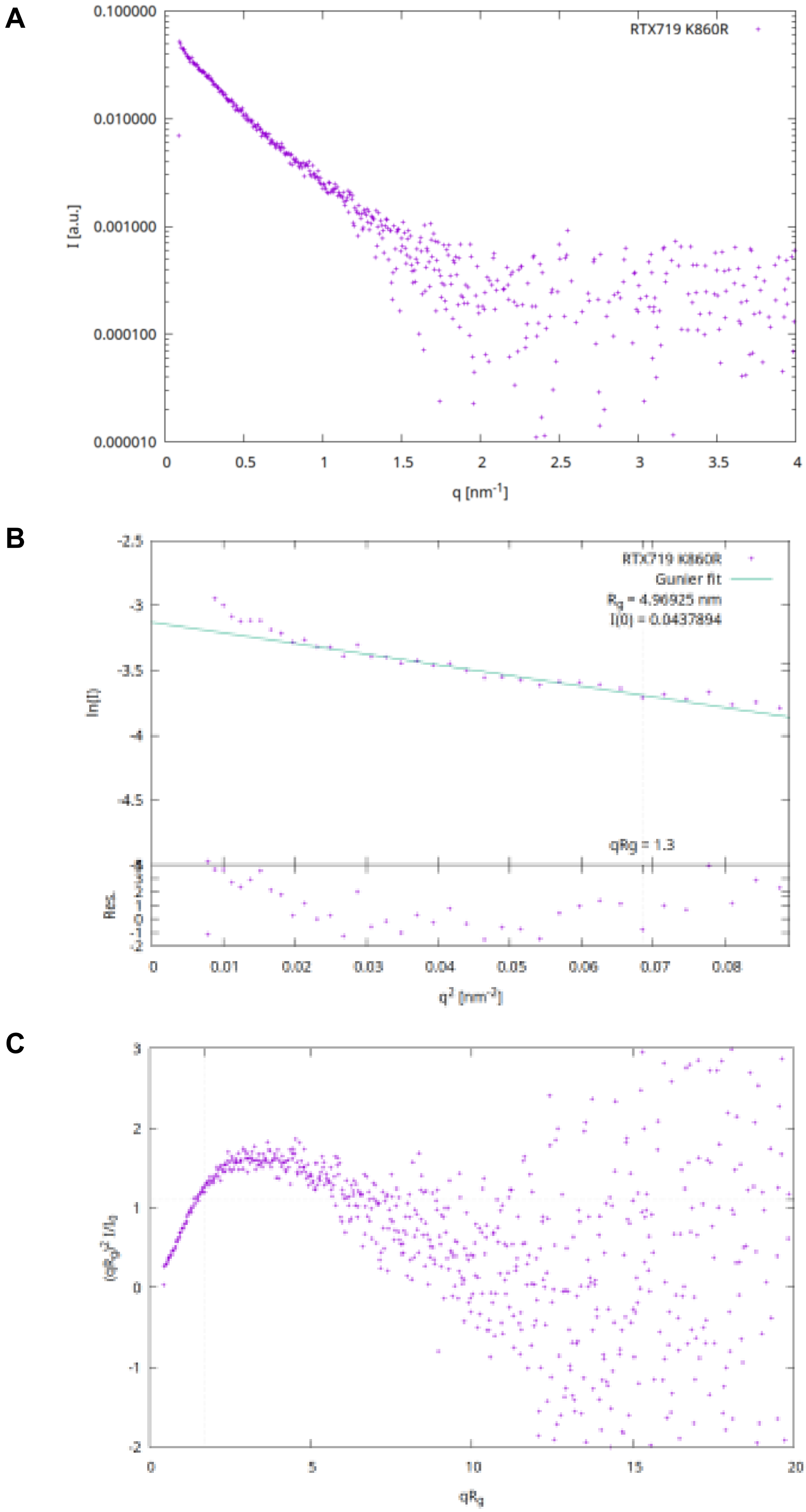
A
RTX719 K860R
I [a.u.]
q [nm⁻¹]
B
RTX719 K860R
Gunier fit
Rg = 4.96925 nm
I(0) = 0.0437894
ln(I)
qRg = 1.3
Res.
q² [nm⁻²]
C
(qRg)² I/I0
qRg

Figure S2 (continued)

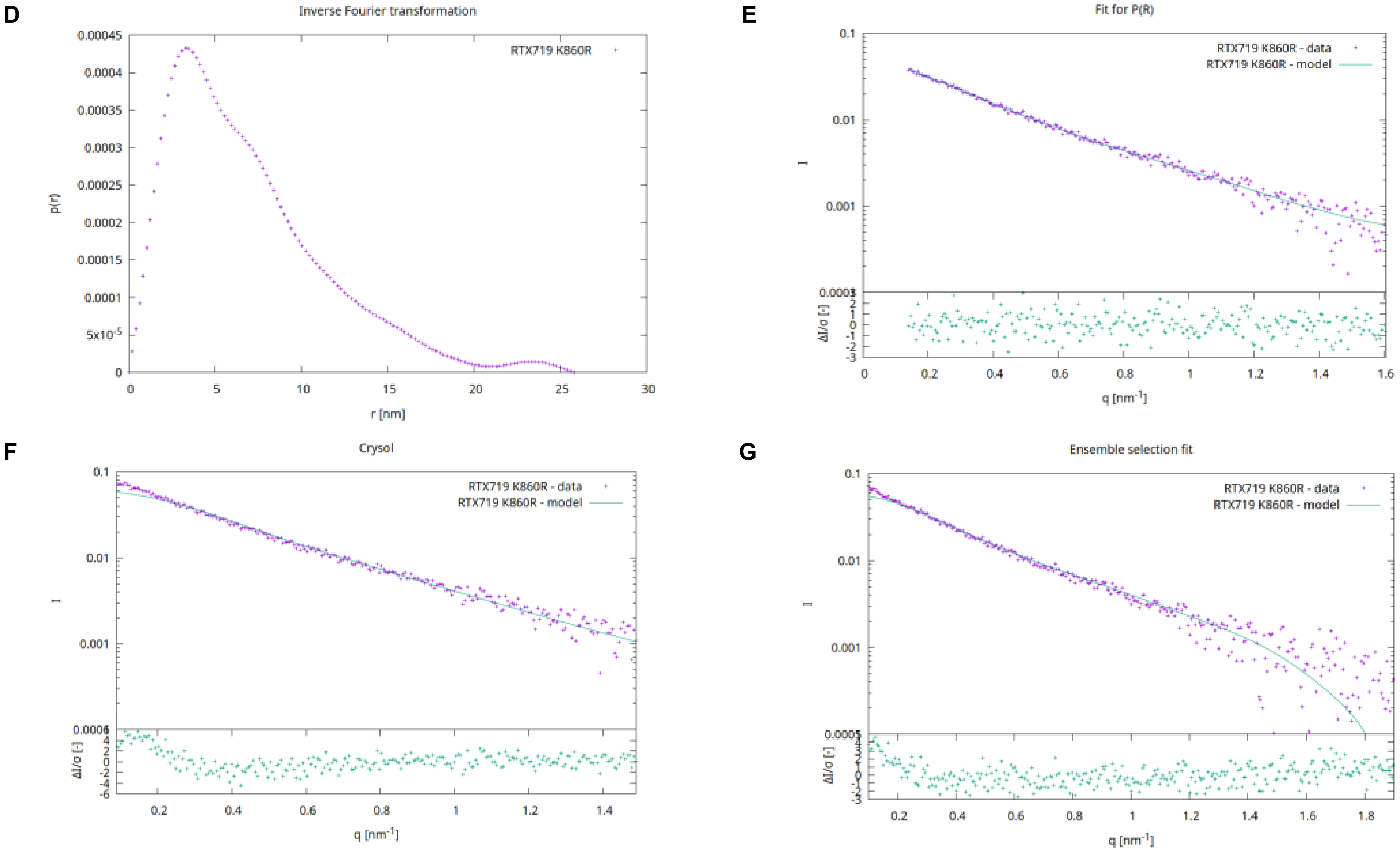

Figure S2 (continued)

H

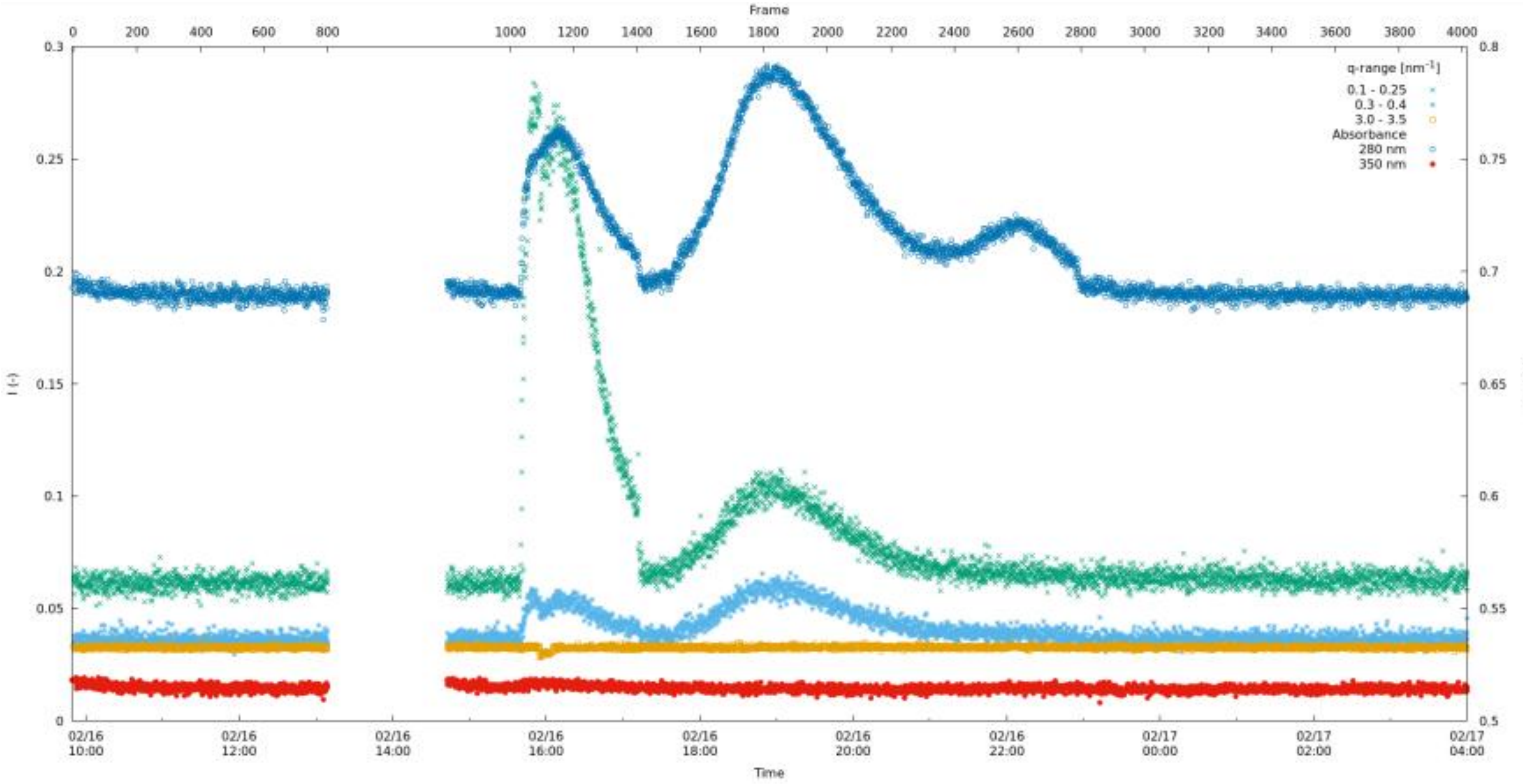

Figure S3

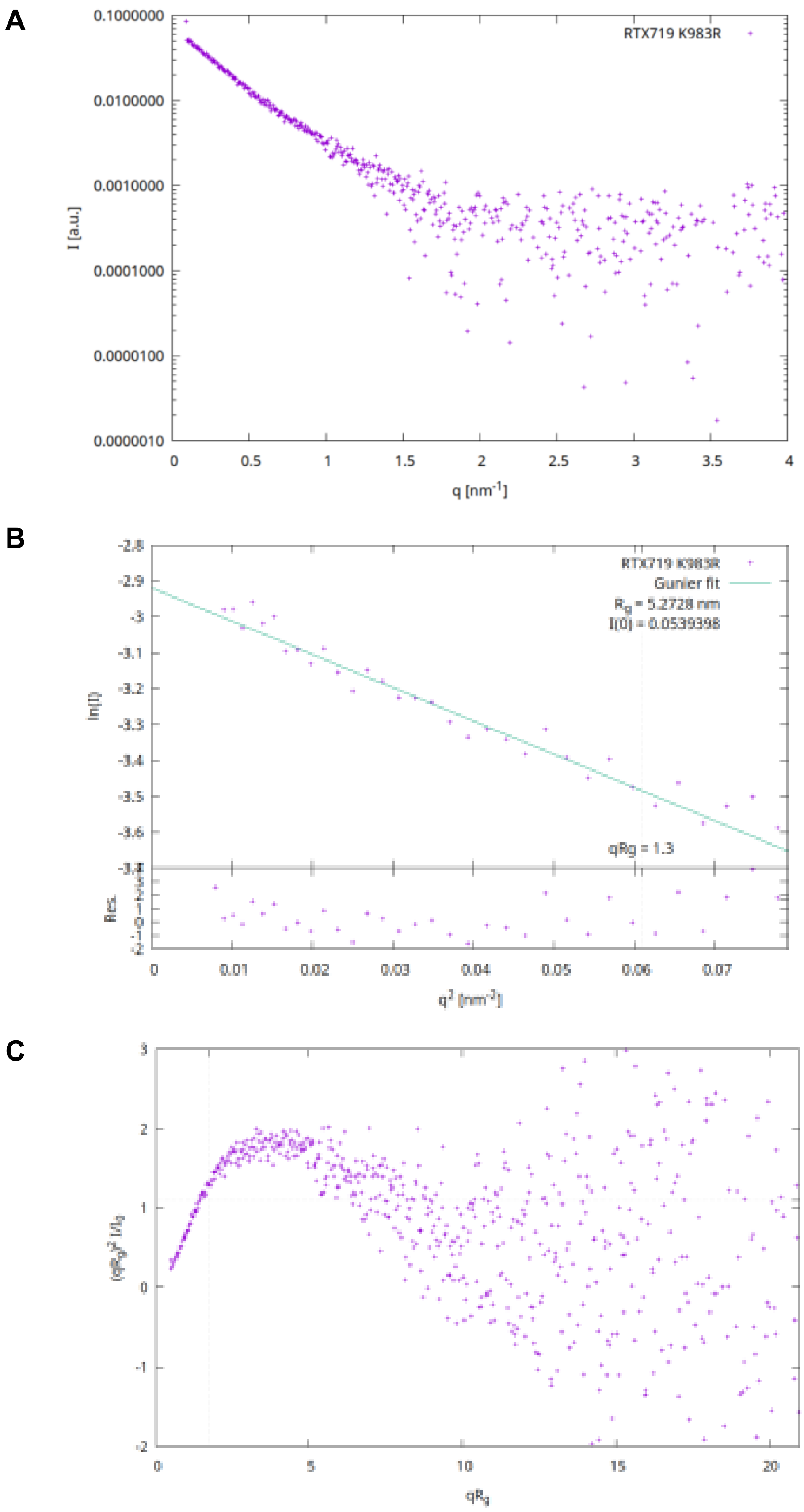

A
RTX719 K983R
I [a.u.]
q [nm-1]
B
RTX719 K983R
Gunier fit
Rg = 5.2728 nm
I(0) = 0.0539398
qRg = 1.3
ln(I)
Res.
q2 [nm-2]
C
(qRg)2 I/I0
qRg

Figure S3 (continued)

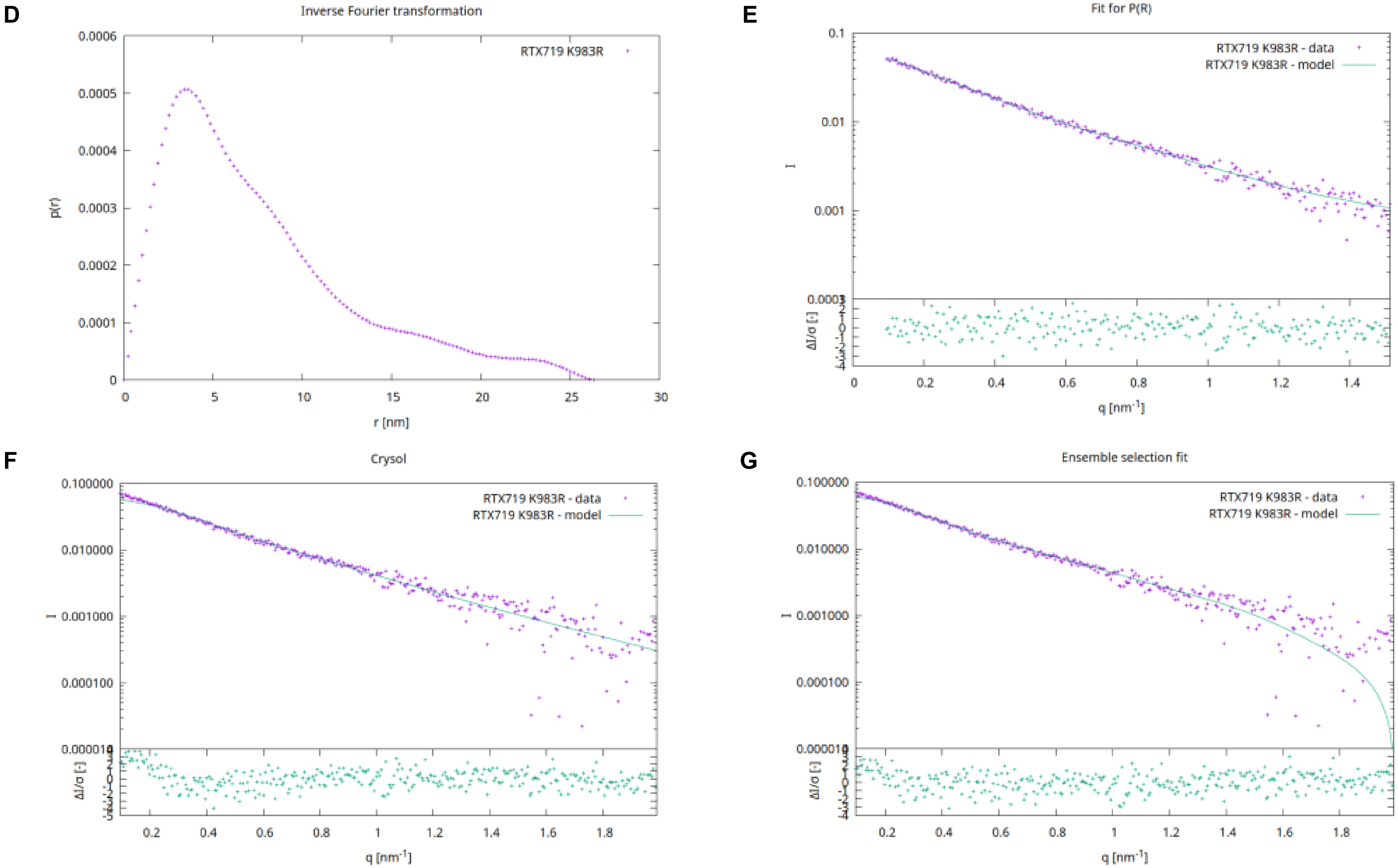

Figure S3 (continued)

H

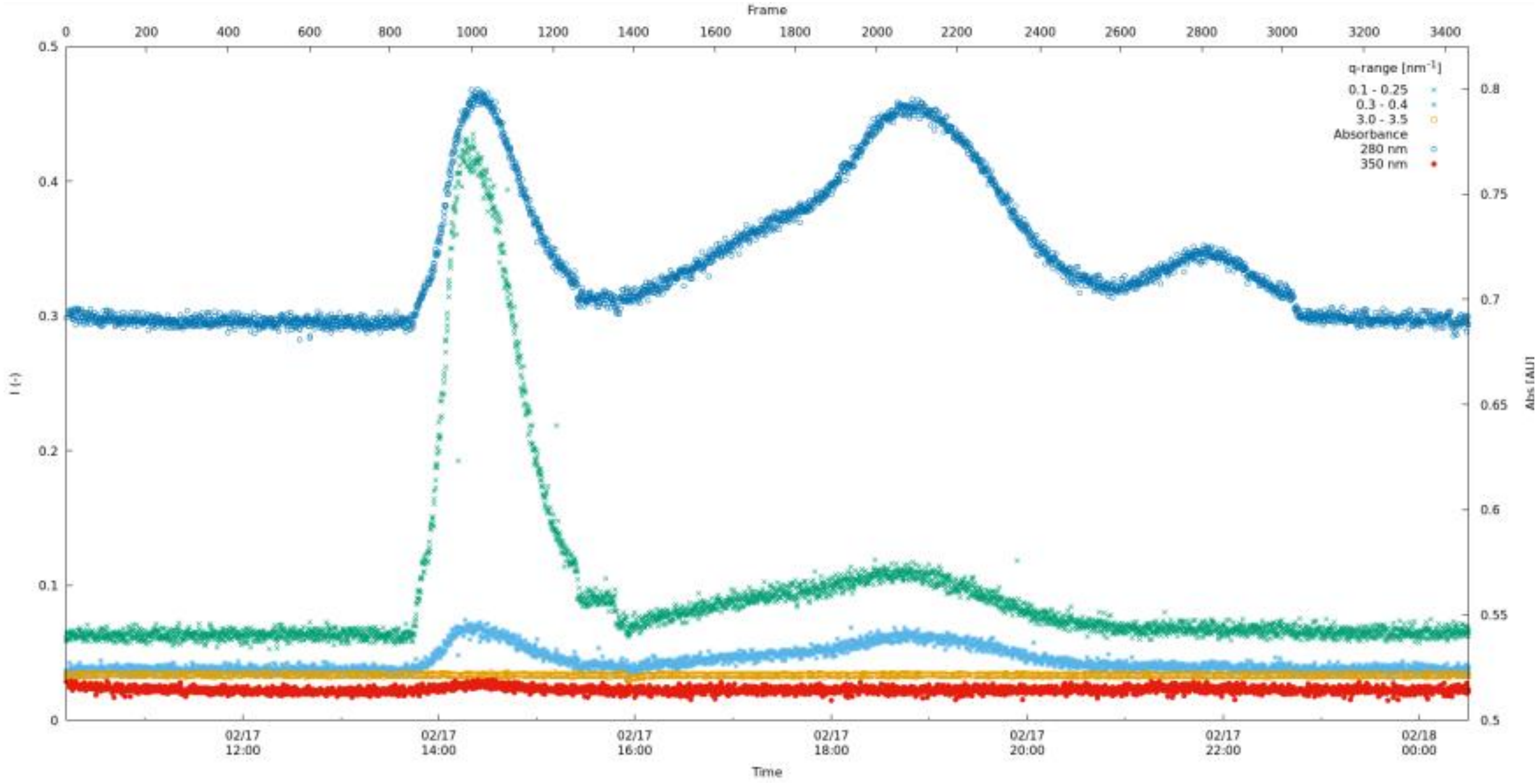

Figure S4

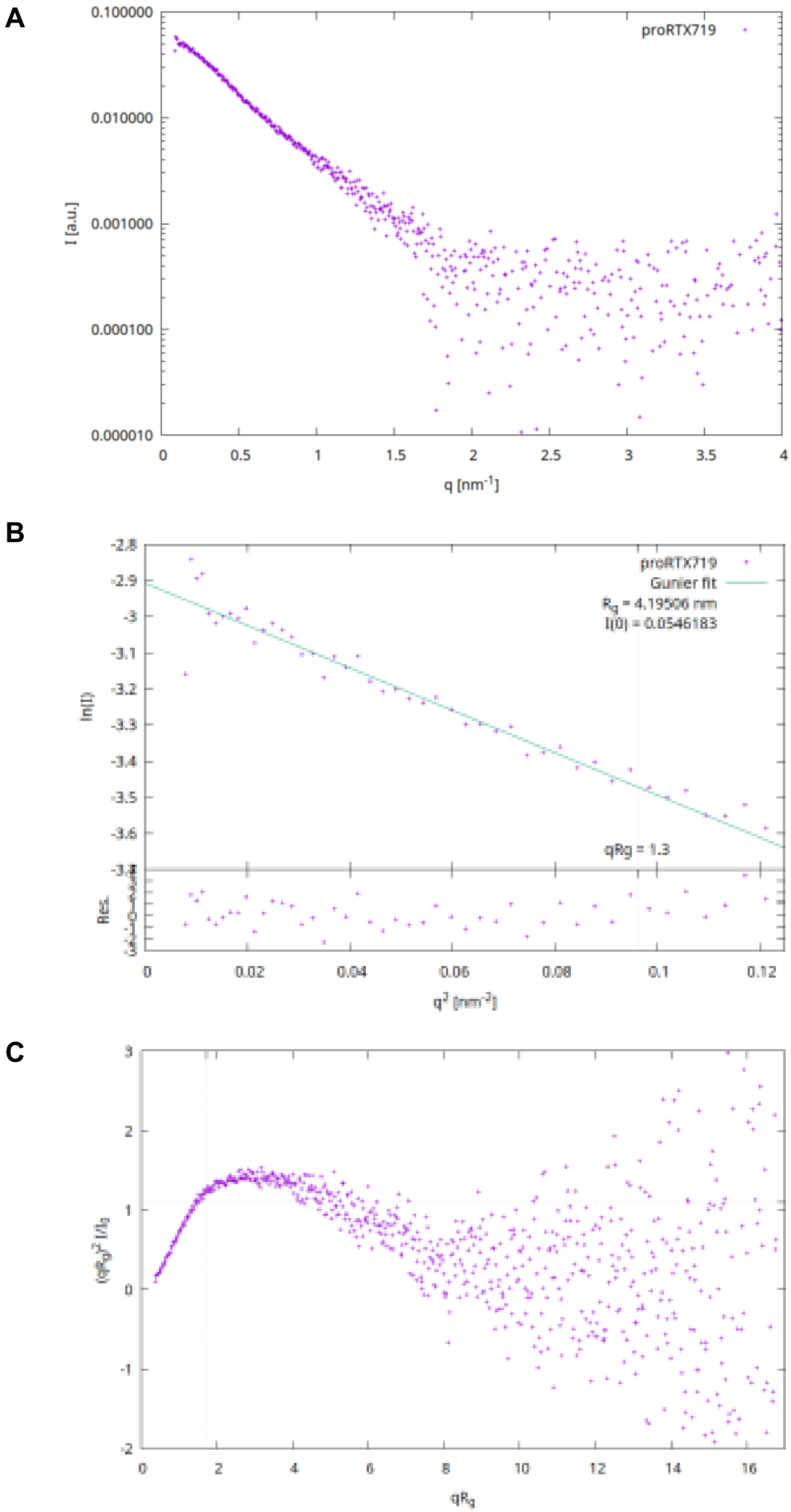
A
0.100000
0.010000
0.001000
0.000100
0.000010
I [a.u.]
q [nm^{-1}]
proRTX719
B
proRTX719
Gunier fit
R_g = 4.19506 nm
I(0) = 0.0546183
qRg = 1.3
ln(I)
Res.
q^2 [nm^{-2}]
C
$(qR_g)^2$ I/I$_0$
qR_g

Figure S4 (continued)

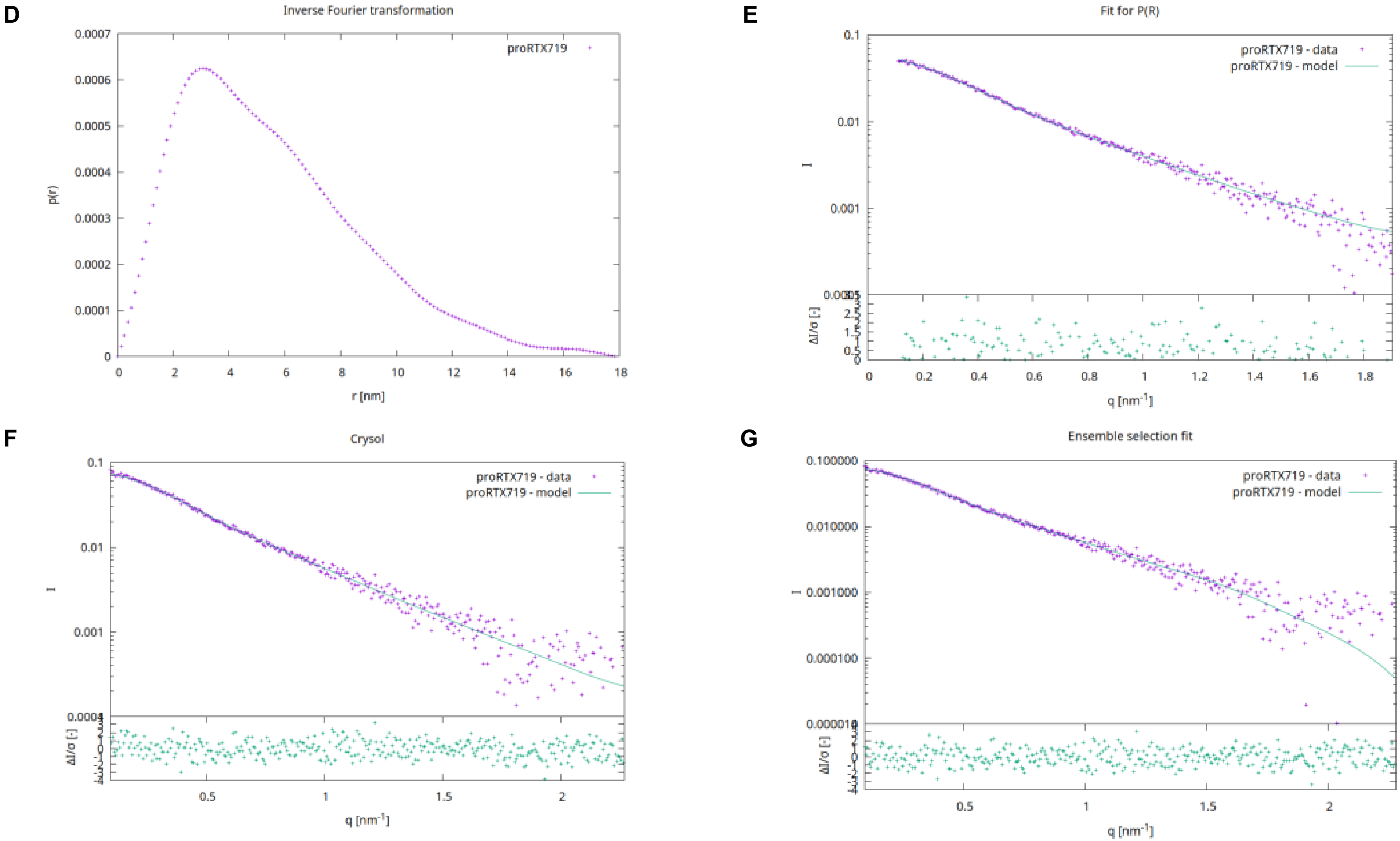

Figure S4 (continued)

H

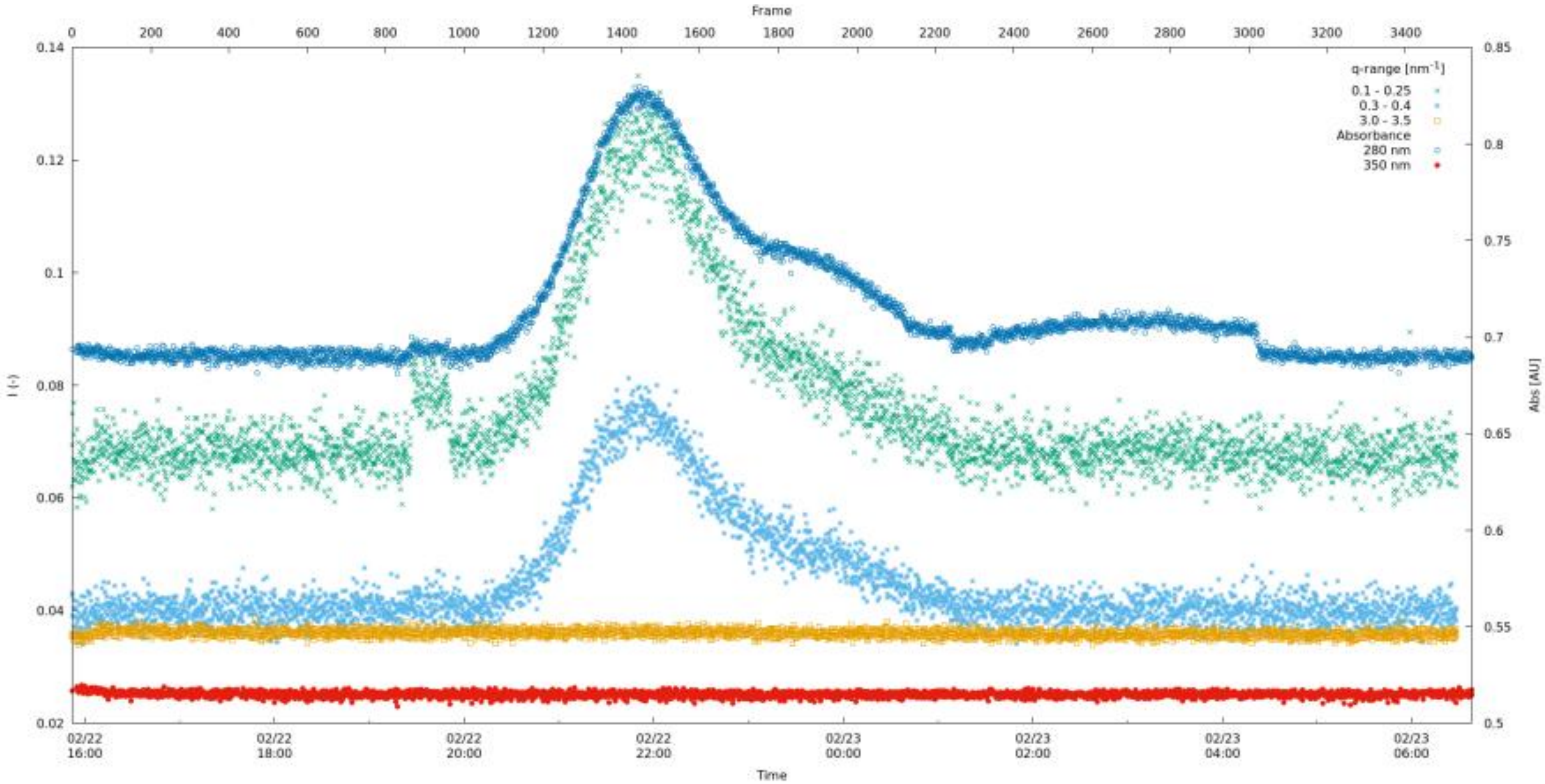

Figure S5

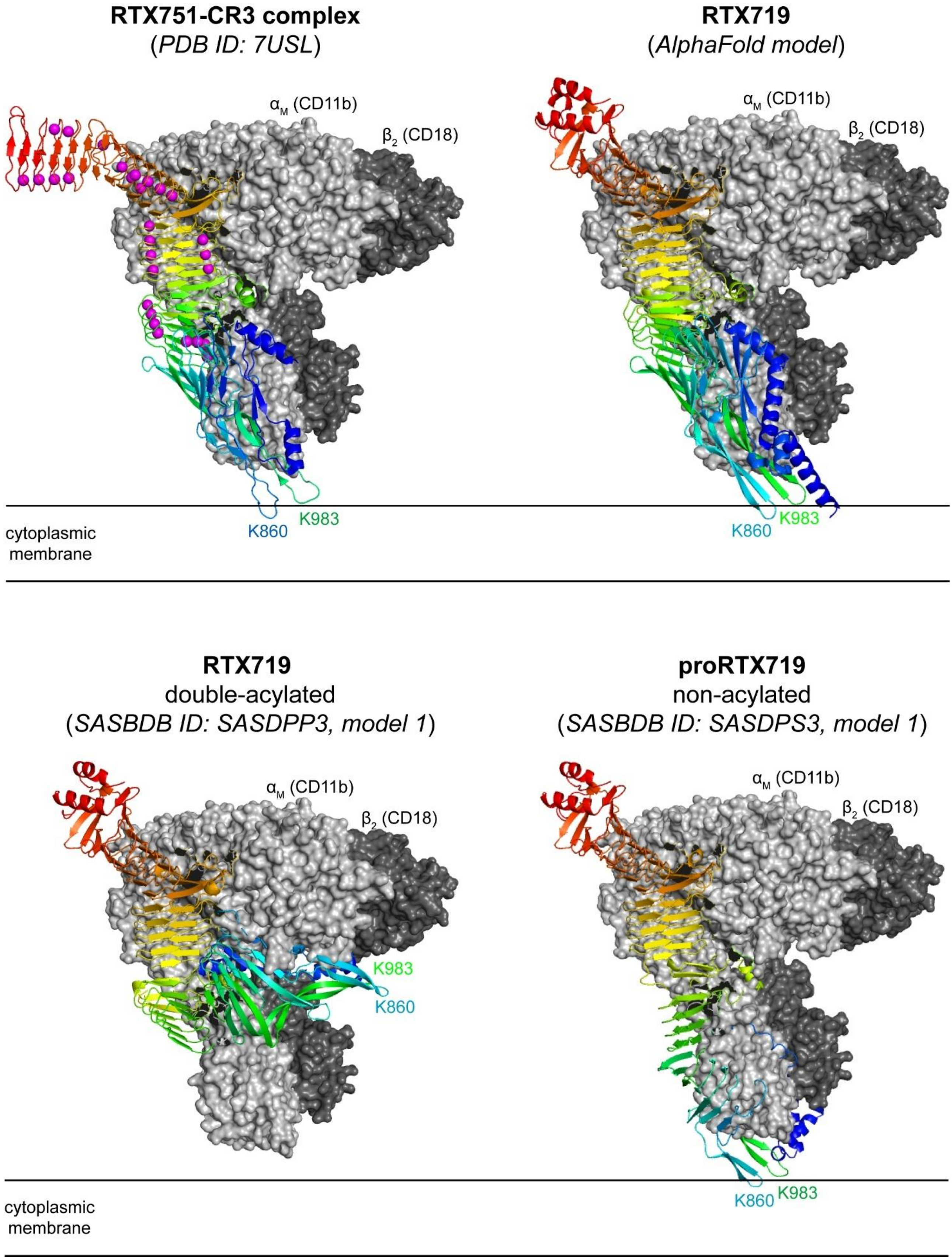
RTX751-CR3 complex
(PDB ID: 7USL)
RTX719
(AlphaFold model)
αM (CD11b)
β2 (CD18)
K860
K983
αM (CD11b)
β2 (CD18)
K860
K983
cytoplasmic
membrane
RTX719
double-acylated
(SASBDB ID: SASDPP3, model 1)
proRTX719
non-acylated
(SASBDB ID: SASDPS3, model 1)
αM (CD11b)
β2 (CD18)
K983
K860
αM (CD11b)
β2 (CD18)
K860
K983
cytoplasmic
membrane
